# Medical records-based chronic kidney disease phenotype for clinical care and “big data” observational and genetic studies

**DOI:** 10.1038/s41746-021-00428-1

**Published:** 2021-04-13

**Authors:** Ning Shang, Atlas Khan, Fernanda Polubriaginof, Francesca Zanoni, Karla Mehl, David Fasel, Paul E. Drawz, Robert J. Carrol, Joshua C. Denny, Matthew A. Hathcock, Adelaide M. Arruda-Olson, Peggy L. Peissig, Richard A. Dart, Murray H. Brilliant, Eric B. Larson, David S. Carrell, Sarah Pendergrass, Shefali Setia Verma, Marylyn D. Ritchie, Barbara Benoit, Vivian S. Gainer, Elizabeth W. Karlson, Adam S. Gordon, Gail P. Jarvik, Ian B. Stanaway, David R. Crosslin, Sumit Mohan, Iuliana Ionita-Laza, Nicholas P. Tatonetti, Ali G. Gharavi, George Hripcsak, Chunhua Weng, Krzysztof Kiryluk

**Affiliations:** 1 Department of Biomedical Informatics, Vagelos College of Physicians & Surgeons, Columbia University, New York, NY USA; 2 Division of Nephrology, Department of Medicine, Vagelos College of Physicians & Surgeons, Columbia University, New York, NY USA; 3 Department of Medicine, University of Minnesota, Minnesota, MN USA; 4 Department of Biomedical Informatics, Vanderbilt University, Nashville, TN USA; 5 Departments of Medicine, Vanderbilt University, Nashville, TN USA; 6 Department of Biomedical Informatics, Mayo Clinic, Rochester, MN USA; 7 Department of Cardiovascular Diseases, Mayo Clinic, Rochester, MN USA; 8 Marshfield Clinic Research Institute, Marshfield, WI USA; 9 Kaiser Permanente Washington Health Research Institute, Seattle, WA USA; 10 Geisinger Research, Rockville, MD USA; 11 University of Pennsylvania, Philadelphia, PA USA; 12 Partners HealthCare, Somerville, MA USA; 13 Harvard Medical School, Harvard University, Cambridge, MA USA; 14 Center for Genetic Medicine, Northwestern University, Chicago, IL USA; 15 Departments of Medicine (Medical Genetics) and Genome Sciences, University of Washington School of Medicine, Seattle, WA USA; 16 Department of Biomedical Informatics and Medical Education, University of Washington, Seattle, WA USA; 17 Department of Biostatistics, Mailman School of Public Health, Columbia University, New York, NY USA

**Keywords:** Chronic kidney disease, Genetics research, Epidemiology

## Abstract

Chronic Kidney Disease (CKD) represents a slowly progressive disorder that is typically silent until late stages, but early intervention can significantly delay its progression. We designed a portable and scalable electronic CKD phenotype to facilitate early disease recognition and empower large-scale observational and genetic studies of kidney traits. The algorithm uses a combination of rule-based and machine-learning methods to automatically place patients on the staging grid of albuminuria by glomerular filtration rate (“A-by-G” grid). We manually validated the algorithm by 451 chart reviews across three medical systems, demonstrating overall positive predictive value of 95% for CKD cases and 97% for healthy controls. Independent case-control validation using 2350 patient records demonstrated diagnostic specificity of 97% and sensitivity of 87%. Application of the phenotype to 1.3 million patients demonstrated that over 80% of CKD cases are undetected using ICD codes alone. We also demonstrated several large-scale applications of the phenotype, including identifying stage-specific kidney disease comorbidities, in silico estimation of kidney trait heritability in thousands of pedigrees reconstructed from medical records, and biobank-based multicenter genome-wide and phenome-wide association studies.

## Introduction

Chronic Kidney Disease (CKD) is associated with a high burden of comorbidities and increased mortality^1,2^. Due to the increasing prevalence, and high costs of renal replacement therapies, CKD already represents one of the most expensive health problems in developed countries^3^. In the United States, an estimated 13.6% of adults have CKD^1^ and more than 726,331 Americans have end-stage kidney disease (ESKD), being dialysis-dependent or having received a kidney transplant^4^. ESKD prevalence is about 3.7 times greater in African Americans, 1.4 times greater in Native Americans, and 1.5 times greater in Asian Americans than in Whites/Europeans. Inherited factors, such as *APOL1* polymorphisms^5,6^ and other genetic factors^7,8^, are likely contributing to these disparities.

CKD is defined as an abnormality of kidney structure or function present for longer than 90 days and can occur due to many heterogeneous disorders affecting the kidney^9,10^. Unlike most other disease states, the onset of kidney disease is often asymptomatic, and the diagnosis is based solely on blood and/or urine tests. As a result, early CKD is frequently under-recognized and under-treated^11^. While several measures, such as dietary interventions, hyperlipidemia management with statins^12^, blood pressure control^13^, glycemic control^14^, and use of angiotensin system blockade^15^ or sodium-glucose cotransporter-2 inhibitors^16,17^ can slow down the progression of early disease or reduce complications, advanced CKD is irreversible and associated with accelerated cardiovascular disease and increased mortality^18^. Thus, early detection and improved awareness of CKD is of paramount importance.

Electronic health records (EHR) provide a rich source of clinical data that can be used reliably to establish a CKD diagnosis. With increased reliance on the EHR for pragmatic implementation of clinical and genetic studies, there is an unmet need for a standardized portable electronic definition of CKD and its severity. To address this need, we designed a comprehensive electronic CKD phenotype that combines expert domain knowledge and the consensus definitions of the National Kidney Foundation’s (NKF) Kidney Disease Outcomes Quality Initiative (KDOQI) guidelines^19^ and the Kidney Disease: Improving Global Outcomes (KDIGO) Clinical Practice Guideline for the Evaluation and Management of CKD^9,10^. We designed our algorithm to detect CKD in its earliest stages by calculating two orthogonal measures of CKD severity: albuminuria (used for A-staging of CKD) and estimated glomerular filtration rate (eGFR, used for G-staging of CKD).

Our electronic phenotyping approach provides pragmatic means to enhance broad and proactive CKD screening, risk stratification, and timely initiation of treatment to reduce the global burden of kidney disease, as recommended by the 2019 consensus statement of the KDIGO Conference on “Early Identification and Intervention in CKD”^20^. To assure transferability across different EHR systems, our algorithm was developed using training and validation datasets across several institutions, including Columbia University (CU), University of Minnesota (UMN), Vanderbilt University (VU), and Mayo Clinic (MC). To show scalability and portability, the algorithm was applied to the Columbia Clinical Data Warehouse (CDW) of over 1.3 million patients, as well as across the entire Electronic Medical Records and Genomics-III (eMERGE-III) network of eight centers with genetic and EHR data for 105,108 individuals^21^.

We demonstrated several powerful applications of the algorithm. First, we performed large scale observational analyses of common comorbidities across the A-by-G grid to define independent associations for A and G-stage, including several comorbidity patterns that have not been previously recognized. Second, we applied our recently published Relationship Inference From The Electronic Health Record (RIFTEHR) method to computationally infer familial relationships from EHR data and estimate pedigree-based observational heritability of kidney disease^22^. Using thousands of reconstructed pedigrees of diverse ancestries, we demonstrated significant heritability of eGFR, albuminuria, and CKD at a scale previously unobtainable for family-based studies. Third, we performed genome-wide association studies for CKD across the eMERGE network, demonstrating that our algorithmic phenotype definition recovers known genome-wide significant risk loci. Finally, we analyzed 19,853 distinct ICD codes mapped to 1804 phecodes in all 105,108 eMERGE participants to comprehensively define pleiotropic associations of the top CKD risk loci using phenome-wide association approach^23^.

In summary, we created an accurate, portable, and scalable electronic phenotype for CKD diagnosis and staging. We performed extensive validations of the algorithm and demonstrated its broad clinical and research applications, from enabling automated detection of patients that would benefit from renoprotective therapies, to empowering “big data” genetic and observational studies of CKD at a scale unobtainable using traditional phenotyping methods.

## Results

### Design and performance of electronic CKD phenotype

**Fig. 1:**
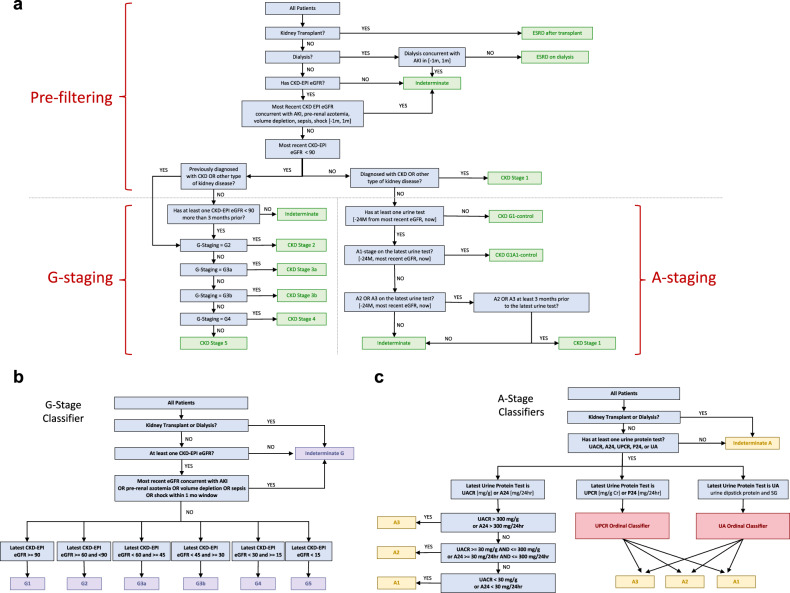
Electronic CKD diagnosis and staging algorithm. **a** Flowchart of the National Kidney Foundation (NKF) criteria-based algorithm composed of three parts: data pre-filtering, G-staging, and A-staging **b** G-Stage Classifier for staging of CKD based on estimated glomerular filtration rate (eGFR), and **c** A-Stage Classifiers for staging of CKD based on albuminuria. UACR Urine Albumin-to-Creatinine Ratio, UPCR Urine Protein-to-Creatinine Ratio, A24 24-h urine collection for albumin, P24 24-h urine collection for protein, UA Urinalysis, SG Specific Gravity.

We describe the details of algorithm development and validation in the Methods section. Briefly, we combined the NKF KDOQI guidelines^19^, the KDIGO Clinical Practice Guideline for the Evaluation and Management of CKD^9,10^, and domain expert knowledge in nephrology to define CKD cases and controls using laboratory measurements in combination with diagnosis and procedure codes (Fig. 1). Any patient with relevant EHR data is staged based on eGFR (G-stage) and albuminuria (A-stage). To accomplish G-staging, we designed a rule-based “G-Stage Classifier” that uses thresholding based on the most recent qualifying eGFR. We performed A-staging using a set of “A-Stage Classifiers”, which we based on the most recent urine protein or albumin tests. We employed supervised machine-learning approaches to harmonize A-stage classification across all commonly ordered urine tests.

We provide an overall flowchart summary of the CKD phenotype in Fig. 1a. After using diagnostic and procedure codes to first identify and categorize patients with ESRD on dialysis or those with a kidney transplant, the algorithm uses rule-based filters to eliminate lab values measured concurrently with acute conditions known to impact the steady state of creatinine clearance. We then use the most recent serum Cr value to estimate GFR. The algorithm accounts for disease chronicity by requiring either a pre-existing billing code consistent with a CKD diagnosis or another qualifying eGFR or proteinuria value at least 90 days before to the value used for staging. The G-stage and A-stage classifiers are then applied to accomplish the staging.

The G-stage classifier (Fig. 1b) requires several rule-based pre-filtering steps followed by a simple threshold-based G-stage classification using the latest qualifying eGFR. The A-stage classification problem (Fig. 1c) needed a machine-learning solution to harmonize classifications between different proteinuria measurements. Using several real-life training datasets from three major US medical centers, we developed an exhaustive set of albuminuria classifiers that could accommodate all commonly performed clinical urine protein quantification tests (see Methods and Supplementary Tables 1–5). We then used cross-validation studies to create a “preference ranking” of proteinuria tests based their relative classification performance (Table 1). From high to low, the preference rankings included UACR or 24-h urine collection for albumin (direct measurement, “gold standard”), UPCR or 24-h urine collection for protein (80–92% accuracy), to DSP with urine specific gravity (76–95% accuracy). We incorporated the preference order into the algorithm’s workflow.

**Table 1:** Overall performance of the A-stage classifiers used in the algorithm.

Test	UPCR-based A-Stage Classifier	DSP-based (Scale 1) A-Stage Classifier	DSP-based (Scale 2) A-Stage Classifier
	*n* = 19,099 paired measurements	*n* = 12,185 paired measurements	*n* = 43,486 paired measurements
Squared error	0.219 (0.213, 0.224)	0.256 (0.251, 0.261)	0.235 (0.23, 0.241)
Accuracy (95% CI)			
A1	86.7% (86.4%, 87.0%)	80.9% (80.5%, 81.3%)	82.2% (81.8%, 82.5%)
A2	80.0% (79.7%, 80.3%)	76.0% (75.6%, 76.4%)	78.5% (78.1%, 79.0%)
A3	92.3% (92.0%, 92.6%)	94.3% (94.1%, 94.4%)	95.3% (95.1%, 95.5%)
Sensitivity (95% CI)			
A1	86.2% (85.2%, 87.1%)	90.9% (89.9%, 91.9%)	93.2% (93.0%, 93.4%)
A2	63.5% (62.4%, 64.5%)	41.4% (40.1%, 42.8%)	35.7% (34.0%, 37.4%)
A3	86.7% (85.9%, 87.5%)	83.3% (82.1%, 84.4%)	81.0% (80.0%, 82.0%)
Specificity (95% CI)			
A1	87.1% (86.4%, 87.7%)	69.6% (68.6%, 70.5%)	62.1% (61.1%, 63.1%)
A2	87.1% (86.6%, 87.5%)	89.5% (88.7%, 90.2%)	92.0% (91.9%, 92.2%)
A3	94.8% (94.5%, 95.1%)	96.8% (96.5%, 97.1%)	97.1% (97.0%, 97.3%)

The 95% confidence intervals were calculated based on 10-fold cross-validation; DSP Scale 1: reported as Negative, Trace, 1 + , 2 + , 3 + , 4 + ; DSP Scale 2: reported as Negative, Trace, 10, 30, 100, 300, or ≥ 300; only the performance of the final pooled classifiers across Columbia University, University of Minnesota, and Vanderbilt University are summarized, for detailed breakdown of site-specific performance see Tables S1–S5, and for additional validation studies and comparisons with recently published methods by Sumida et al.^24^, see Table S6.

To further test our A-stage prediction, we compared the performance of our classifiers to the alternative methods developed more recently by the CKD Prognosis Consortium^24^ (Supplementary Table 6). Based on an independent testing dataset, we demonstrated that the performance of the two methods was generally comparable. While the UPCR-based classifier developed by the CKD Prognosis Consortium performed slightly better compared to the one developed in our study (overall accuracy of 83% vs. 77%), our urinalysis-based classifiers outperformed the ones developed by the CKD Prognosis Consortium (overall accuracy of 71% vs. 65–67%, respectively). Notably, the urinalysis-based equations developed by the CKD Prognosis Consortium do not account for urine specific gravity, or scale differences in protein dipstick tests, potentially explaining poorer performance compared to our model.

To validate the performance of our CKD detection and staging algorithm, we determined the overall and stage-specific positive predictive values (PPV) of the algorithmic diagnoses by performing 451 blinded manual chart reviews of algorithm-derived diagnostic labels across three major US medical centers (Table 2). The overall diagnostic PPV was 95% (range 83–99%) for CKD cases and 97% (range 95–100%) for healthy controls.

**Table 2:** Manual validation of the CKD diagnosis and staging algorithm.

Group	Columbia University		Vanderbilt University		Mayo Clinic		Combined	
	N Reviewed	PPV	N Reviewed	PPV	N Reviewed	PPV	N Reviewed	PPV
Controls	62	0.968	20	0.950	20	1.000	102	0.971
Cases	189	0.995	80	0.825	80	0.950	349	0.946
CKD Stage 1	20	0.900	10	0.600	10	1.000	40	0.850
CKD Stage 2	20	1.000	10	1.000	10	1.000	40	1.000
CKD Stage 3a	20	1.000	10	1.000	10	1.000	40	1.000
CKD Stage 3b	22	1.000	10	0.800	10	1.000	42	0.952
CKD Stage 4	23	0.913	10	1.000	10	1.000	43	0.953
CKD Stage 5	20	0.750	10	0.200	10	1.000	40	0.675
ESRD after transplant	24	0.792	10	1.000	10	0.800	44	0.818
ESRD on dialysis	40	0.700	10	1.000	10	0.900	60	0.783

The validations were performed by selecting 451 algorithm-defined cases and controls across all stages for blinded chart reviews by domain experts across the three independent validation sites: Columbia University, Vanderbilt University, and Mayo Clinic. We derived positive predictive values (PPVs) for controls and CKD cases combined and by disease stage. The overall diagnostic PPV was 95% (range 83–99%) for CKD cases and 97% (range 95–100%) for healthy controls.

To perform additional testing of the algorithm and to enable estimation of the overall diagnostic sensitivity and specificity, we constructed a large case-control dataset of 2350 patients. We defined 1136 cases as patients seen by a nephrologist in the Columbia CKD clinic, and 1214 controls as healthy women without a known CKD diagnosis undergoing a prenatal visit at Columbia University during the same time interval as the cases. In this dataset, the sensitivity, specificity, PPV and NPV of the algorithm were 87%, 97%, 97%, and 89%, respectively (Supplementary Table 7). Notably, the algorithm identified no cases of CKD stage 3 or greater among patients seen in the prenatal clinic and did not call a single non-CKD control among the CKD clinic patients. While high specificity of 97% reflects the fact that our algorithm uses a stringent set of diagnostic criteria, lower sensitivity of 87% is predominantly due to a small fraction of cases with insufficient longitudinal data to meet the chronicity criteria.

We provide an open-source implementation of a parameterized and modularized version of this algorithm through the publicly accessible Phenotype Knowledgebase (https://phekb.org/phenotype/chronic-kidney-disease)^25^. The PheKB documentation also includes a detailed list of all ICD-9-CM, ICD-10-CM, SNOMED, lab LOINC, and procedure CPT codes used by the algorithm.

### Medical records-based observational study of CKD comorbidities

We applied our algorithm to 1,365,098 CUIMC patients with at least one available serum Cr test in their EHR. The algorithm successfully staged 672,858 individuals using the NKF criteria, identifying 13,930 CKD stage I patients, 205,887 CKD stages II–V (non-dialysis) patients, and 19,515 ESRD patients on dialysis or after kidney transplant. Notably, only 45,405 (19%) of algorithm-diagnosed CKD cases had a diagnostic or procedure code compatible with CKD, demonstrating superior sensitivity of our phenotyping approach. The counts by the NKF stage and KDIGO A-by-G grid are provided in Supplementary Table 8.

**Fig. 2:**
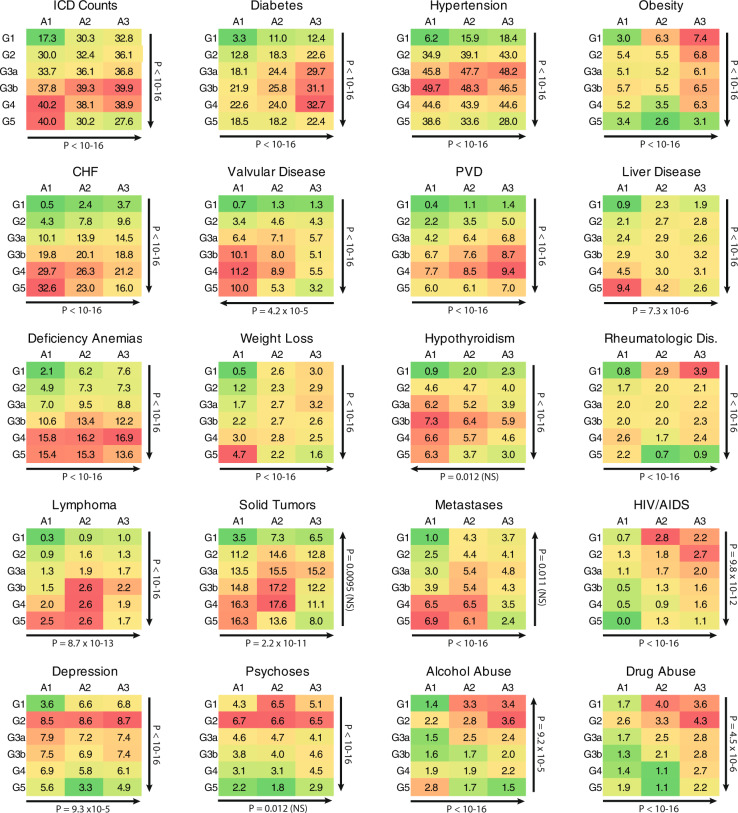
Comorbidity heatmaps for 239,332 CUIMC patients algorithmically placed on the A-by-G grid. The prevalence of a comorbidity within each cell is provided, with the shaded color scale varying from red (highest prevalence) to green (lowest prevalence). The arrows correspond to the direction of effect and *P* values the statistical tests of comorbidity gradients across the grid. The analysis excludes individuals with missing urine tests and those with ESRD on dialysis or after transplant. Models based on logistic regression was used for binary traits and Poisson regression for ICD counts. All models were adjusted for age and sex and *P* value <6.25 × 10^−4^ is considered as significant after Bonferroni correction. NS not significant.

Next, we calculated the prevalence of comorbidities for each NKF stage (Supplementary Table 9), as well as for each cell on the KDIGO A-by-G grid (Fig. 2). Consistent with the existing literature, we detected increasing trends in age and sex-adjusted prevalence for multiple comorbidities associated with each NKF stage (Supplementary Table 9). Our algorithm’s unique A-by-G staging feature allowed us to test for independent effects of A and G stage on the overall burden of comorbid conditions. We first assessed the average number of unique ICD codes per patient. While non-CKD (A1G1) individuals carry an average of 17 unique codes (Fig. 2, top left), this number increased independently with a higher A and G stage. For example, non-albuminuric patients with CKD stage 5 (A1G5) carried an average of 40 unique ICD codes. In comparison, severely albuminuric patients with preserved renal function (A3G1) had an average count of 33 codes. Both A and G stages were independently predictive of the ICD code burden when tested using Poisson regression after controlling for age and sex.

Similarly, we tested for significant patterns in age and sex-adjusted comorbidities defined by the AHRQ Elixhauser Comorbidity Index^26,27^. We observed that a higher A-stage was associated with increased prevalence of the following comorbid conditions, independently of G-stage: diabetes, hypertension, obesity, congestive heart failure, peripheral vascular disease, liver disease, deficiency anemias, weight loss, rheumatologic diseases, lymphomas, solid tumors, metastatic tumors, HIV/AIDS, depression, and drug abuse (Fig. 2).

Many of these conditions represent either a risk factor for, or a consequence of a kidney disease. For example, a strong association of HIV/AIDS with higher A-stage may reflect greater risk of a glomerular disease, such as HIV-associated nephropathy, or exposure to potentially tubulo-toxic protease inhibitors. Similarly, strong associations of solid and hematologic malignancies with A-stage independently of G-stage may represent glomerular complications of malignancies or chemotherapy-related side effects.

We also observed several new or unexpected trends that exceeded our Bonferroni-corrected significance threshold. For example, valvular diseases were positively correlated with G-stage (*P* < 1 × 10^−16^) as previously recognized^28^ but were also negatively correlated with A-stage (*P* = 4.2 × 10^−5^) after accounting for G-stage, highlighting a new protective association that should be studied. Conversely, alcohol abuse was positively correlated with A-stage (*P* < 1 × 10^−16^) but appeared progressively less common with increasing G-stage (*P* = 9.2 × 10^−5^).

We also noted that several psychiatric comorbidities, including depression, psychoses, and substance abuse (alcohol and drugs), were considerably more prevalent among patients with mild CKD (G2) than patients with normal renal function in age and sex-independent manner. The relationship between CKD and neuropsychiatric diseases has previously been observed in advanced CKD^29^, but a higher risk at early stages has not been previously reported. In summary, we provided a comprehensive landscape of CKD comorbidities and demonstrated the utility of EHR in uncovering new patterns and subpopulations that can be used for targeted interventions.

### Medical records-based observational heritability of CKD

Using emergency contact information, we have previously inferred 3,244,380 unique familial relationships that were used to reconstruct 223,307 pedigrees among patients with EHR records at CUIMC^22^. We intersected these data with our CKD algorithm’s output to estimate pedigree-based observational heritability (h_o_^2^) of renal function. We note that h_o_^2^ is an estimate of the narrow-sense heritability based on observational data. Because observational data are subject to confounding by physician and patient behaviors that may affect the probability that a particular trait is ascertained, we used repeated subsampling with SOLARStrap to produce heritability estimates that are more robust to this bias, as previously described^22^. We also control for age, sex, race/ethnicity, and household effects (see Methods).

**Fig. 3:**
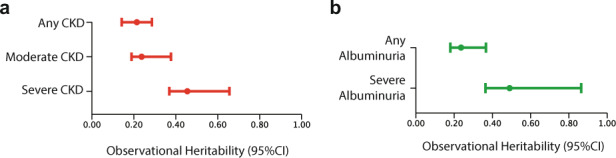
EHR-based observational heritability (h_o_^2^) of renal function and albuminuria. **a** h_o_^2^ of eGFR (quantitative trait) in families with any CKD, moderate CKD (G-stage 3 or greater) and advanced CKD (G-stage 4 or greater) **b** h_o_^2^ of albuminuria (A2 and A3, dichotomous) and severe albuminuria (A3, dichotomous). Bars correspond to 95% confidence intervals around the point estimates.

Our analysis strongly supported significant genetic contributions to renal function (Fig. 3a). Based on 2623 pedigrees with adequate phenotype data, we estimated the overall observational heritability of eGFR at 0.214 (95% CI: 0.142–0.286, *P* = 4.3 × 10^−5^). After stratifying by self-reported ancestry, the h_o_^2^ was 0.244 (95% CI: 0.167–0.377, *P* = 0.013) for African Americans and 0.197 (95% CI: 0.131–0.321, *P* = 0.0071) for White/Europeans. We also estimated h_o_^2^ for eGFR by restricting the pedigrees to those with at least one member with CKD Stage 3 (moderate CKD or greater) and those with at least one member with CKD Stage 4 (advanced CKD or greater). With this ascertainment, the eGFR h_o_^2^ increased to 0.237 (95% CI: 0.189–0.377, *P* = 2.0 × 10^−2^) for moderate CKD, and 0.455 (95% CI: 0.369–0.656, *P* = 9.6 × 10^−3^) for advanced CKD.

Using the liability threshold model^30^, we next analyzed a dichotomous CKD phenotype (any stage) as defined by our algorithm. In the analysis of 3460 pedigrees, we confirmed significant CKD h_o_^2^ at 0.290 (95% CI 0.211–0.410, *P* = 4.2 × 10^−6^). Additional h_o_^2^ estimates stratified by stage and race/ethnicity are provided in Table 3, demonstrating that the heritability was consistently higher for African Americans when compared to other ancestral groups and increases with kidney disease severity.

**Table 3:** EHR-based observational heritability (h_o_^2^) of eGFR, CKD, and albuminuria.

	Number families	h_o_^2^	95L	95U	SE	*P*	Number attempts	Number converged	Number significant	POSA
**Estimated GFR**										
Any CKD	2623	0.214	0.142	0.286	0.054	4.3E-05	200	200	200	1.00
Any CKD White	919	0.197	0.131	0.321	0.080	7.1E-03	200	197	152	0.77
Any CKD Black	459	0.244	0.167	0.377	0.109	1.3E-02	200	196	152	0.78
Moderate CKD	456	0.237	0.189	0.377	0.115	2.0E-02	200	139	16	0.12
Advanced CKD	131	0.455	0.369	0.656	0.186	9.6E-03	200	107	10	0.09
**Albuminuria**										
Any albuminuria	1122	0.236	0.180	0.366	0.439	1.8E-02	200	181	53	0.29
Severe albuminuria	417	0.490	0.364	0.864	0.313	1.5E-02	200	171	73	0.43
**Any CKD**										
All	3460	0.290	0.211	0.410	0.064	4.2E-06	200	5	5	1.00
Hispanic	3136	0.251	0.185	0.296	0.091	3.1E-05	200	15	15	1.00
White	977	0.323	0.234	0.515	0.114	7.8E-03	200	197	137	0.70
Black	433	0.435	0.291	0.657	0.187	1.3E-02	200	195	128	0.66
**Moderate CKD**										
All	1529	0.618	0.418	0.822	0.089	1.1E-08	200	197	197	1.00
White	310	0.555	0.315	0.845	0.411	9.1E-03	200	199	174	0.87
Hispanic	1024	0.513	0.298	0.781	0.251	6.3E-05	200	199	197	0.99
Black	174	0.777	0.544	0.988	0.351	3.4E-02	200	142	87	0.61
**Advanced CKD**										
All	537	0.761	0.512	0.964	0.086	7.0E-07	200	186	186	1.00
White	112	0.639	0.398	0.928	0.263	3.7E-02	200	186	128	0.69
Hispanic	344	0.727	0.434	0.979	0.322	2.3E-03	200	175	170	0.97
Black	70	0.815	0.540	0.993	0.226	1.0E-02	200	79	30	0.38

The estimates were based on the pedigrees built with RIFTEHR (Relationship Inference From The Electronic Health Record). Estimated GFR was modeled as a quantitative trait, while albuminuria and CKD as dichotomous traits. The numbers of families with available phenotypes is provided, race/ethnicity was determined by self-report for >50% relatives per pedigree. All estimates are adjusted for age, sex, race/ethnicity, and common environment. The SOLARStrap algorithm was run 200 times, subsampling 15-30% of families per run. We used the proportion of significant attempts (POSA) as a quality score for the heritability estimates generated by SOLAR*Strap* as described by Polubriaginof et al. (*Cell*, 2018). h_o_^2^: estimated observational heritability; 95L: lower bound of 95% confidence interval for h_o_^2^; 95U: upper bound of 95% confidence interval for h_o_^2^; SE: standard error. Race/ethnicity is defined by self-report and determined by the majority of family members.

The algorithmic A-staging also provided us with an opportunity to estimate h_o_^2^ of albuminuria (Fig. 3b). Based on the analysis of 1122 pedigrees, the h_o_^2^ of any albuminuria (A2 or A3) was estimated at 0.236 (95% CI: 0.180–0.366, *P* = 0.018). For a subset with heavy albuminuria (A3), the h_o_^2^ was 0.490 (95% CI: 0.364–0.864, *P* = 0.015). Because of a smaller number of A-staged pedigrees, we could not further sub-stratify this analysis by ancestry.

Taken together, these analyses provide the largest and most comprehensive pedigree-based analysis of heritability for kidney function, albuminuria, and CKD presently. They are based on a multiethnic urban cohort of the size that was previously unobtainable for family-based analyses. The results are generally consistent with prior estimates based on much smaller pedigree-based studies^8,31–35^, but we also observed higher heritability of kidney disease in African Americans, potentially contributing to the known racial disparities in CKD risk.

### Genome-wide and phenome-wide association studies

**Fig. 4:**
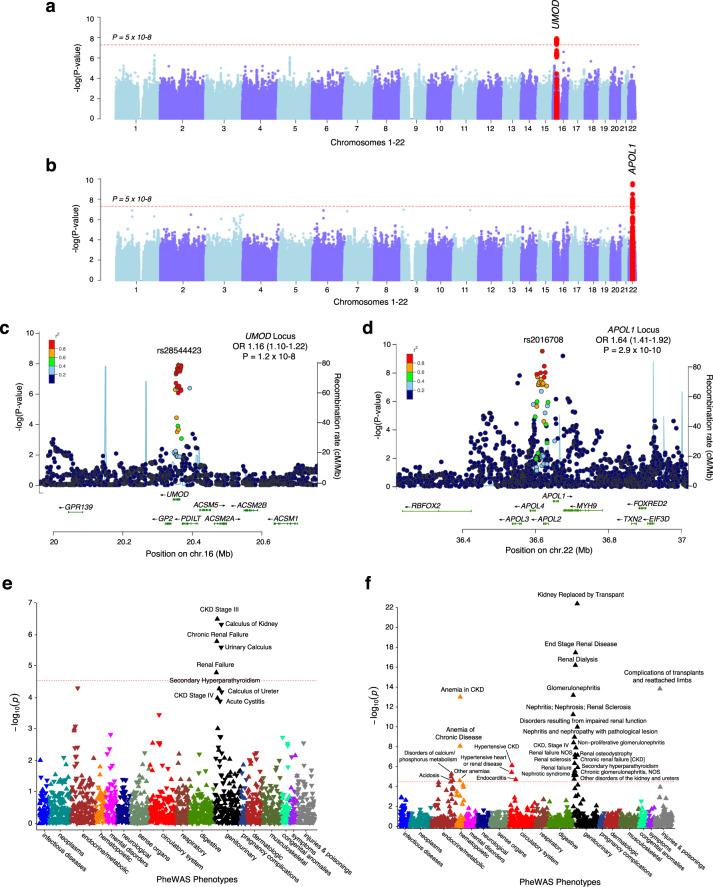
Combined GWAS-PheWAS approach for moderate CKD (G3 or greater). Manhattan plots for **a** eMERGE Europeans (7536 cases, 17,841 controls) with a genome wide-significant signal at the *UMOD* locus (red); **b** eMERGE African-Americans (702 cases, 2029 controls) with a genome wide-significant signal at the *APOL1* locus (red); regional plots for the **c** *UMOD* and **d** *APOL1* loci; eMERGE-based PheWAS plot for the top SNPs at the **e** *UMOD* (*n* = 78,638 Europeans) and **f** *APOL1* (*n* = 16,976 African Americans) loci; upward triangles refer to increased risk; downward triangles indicate reduced risk; horizontal doted lines refer to Bonferroni-corrected significance thresholds.

All sites participating in eMERGE-III network implemented our CKD phenotype, enabling genome-wide association studies (GWAS) stratified by ancestry. To define GWAS cases, we selected CKD Stage 3 or greater (G3-5 and ESRD) based on the observation that moderate CKD had higher h_o_^2^ compared to milder disease. From these, we derived a cohort of 25,377 European participants, consisting of 7536 CKD cases and 17,841 controls matched by platform and genetic ancestry. We performed GWAS under additive genotype coding with adjustment for age, sex, site/platform, and six significant principal components of ancestry (Fig. 4a). We achieved adequate control of genomic inflation (lambda = 1.04). Our analysis detected a genome-wide significant signal at the *UMOD* locus (rs28544423, OR = 1.16, 95% CI: 1.10–1.22, *P* = 1.2 × 10^−8^), a known GWAS risk locus in Europeans (Fig. 4c).

We performed a similar analysis among African ancestry, with 2731 algorithmically defined participants (702 cases and 2029 controls). GWAS was performed under an additive model with adjustment for age, sex, site/platform, and three significant principal components of ancestry, with adequate control of genomic inflation (lambda = 1.03). We detected a genome-wide significant signal at the known *APOL1* locus (rs2016708, OR = 1.64, 95% CI: 1.41–1.92, *P* = 2.9 × 10^−10^) (Fig. 4b, d). The top SNP was in linkage disequilibrium with two known *APOL1* kidney disease risk variants (G1 *r*^2^ = 0.47 and G2 *r*^2^ = 0.12 based on the African populations of the 1000 Genomes Project). Neither G1 nor G2 variants were imputed at high confidence in the eMERGE dataset.

We next performed phenome-wide association studies (PheWAS) for both *UMOD* and *APOL1* loci. The PheWAS for *UMOD* was performed in all 78,638 available European-ancestry eMERGE participants (Fig. 4e) and clearly recovered the association with the phecode “CKD stage III” (OR = 1.14, *P* = 3.1 × 10^−7^). Moreover, we have recovered the previously reported protective associations of the CKD risk variant with nephrolithiasis, including “calculus of kidney” (OR = 0.86, *P* = 4.7 × 10^−7^), “urinary calculus” (OR = 0.88, *P* = 2.5 × 10^−6^), as well as a suggestive protective association for “acute cystitis” (OR = 0.81, *P* = 1.3 × 10^−4^) (Supplementary Data 1**)**. The mechanisms underlying these protective effects are currently not known.

The PheWAS for *APOL1* risk variants was performed in 16,976 individuals of genetically-defined African ancestry and uncovered a broad spectrum of effects with relatively large effect sizes despite simple additive coding used in PheWAS. These associations included kidney transplantation (OR = 2.04, *P* = 4.1 × 10^−23^), end-stage renal disease (OR = 1.60, *P* = 3.4 × 10^−18^), dialysis (OR = 1.70, *P* = 6.2 × 10^−17^), glomerulonephritis (OR = 2.16, *P* = 6.5 × 10^−14^), as well as numerous complications of kidney disease, including anemia (OR = 1.59, *P* = 9.8 × 10^−14^), renal osteodystrophy (OR = 1.66, *P* = 6.9 × 10^−8^) and transplant comorbidities (OR = 1.83, *P* = 1.5 × 10^−14^, Supplementary Data 2).

Lastly, we performed genome-wide estimates of SNP-based heritability for renal function and CKD based on our GWAS, as well as recent studies reported by the CKDGen Consortium^8,36^ (Table 4). Overall, the SNP-based heritability of CKD was consistently low, ranging from 0.4% to 1.5% in Europeans depending on the GWAS study. The estimates were higher for eGFR, ranging from 5.6% to 8.1% in Europeans. The studies of African Americans were of insufficient sample size to derive reliable estimates of SNP-based heritability, and there are no large-scale GWAS available for CKD in other ancestral groups.

**Table 4:** SNP-based Heritability Estimates for CKD and renal function.

Phenotype	Cohort-Ethnicity	Study	N_cases_ /N_controls_	LD Reference	Method	SNP-based Heritability (SE)
CKD	eMERGE-European	Present study	7,536/17,841	1KG-Europeans	LDSC	0.015 (0.010)
	eMERGE-AA	Present study	702/2,029	1KG-Africans	LDSC	0.092 (0.217)
	eMERGE-Transethnic	Present study	8,238/19,870	1KG-All	LDSC	0.044 (0.029)
CKD	CKDGen-European	Wuttke et al.	41,395/439,303	1KG-Europeans	LDSC	0.005 (0.0009)
	CKDGen-Transethnic	Wuttke et al.	64,164/561,055	1KG-All	LDSC	0.004 (0.0008)
	CKDGen-Transethnic	Pattaro et al.	12,385/104,780	1KG-All	LDSC	0.013 (0.004)
eGFR	CKDGen-European	Wuttke et al.	480,698	1KG-Europeans	LDSC	0.056 (0.003)
	CKDGen-Transethnic	Wuttke et al.	765,348	1KG-All	LDSC	0.043 (0.002)
	CKDGen-European	Pattaro et al.	133,814	1KG-Europeans	LDSC	0.081 (0.007)
	CKDGen-AA	Pattaro et al.	16,474	1KG-Africans	LDSC	0.035 (0.045)

We estimated SNP-based heritability of CKD and eGFR from the available genome-wide summary statistics using LDSC method and ancestry-matched linkage disequilibrium reference panels from 1000 Genomes Project (1KG). In addition to present study, we used GWAS summary statistics from the largest published studies of CKD and renal function, including Wuttke et al. (*Nature Genetics*, 2019) and Pattaro et al. (*Nature Communications*, 2016). The summary statistics were downloaded from the CKDGen website (https://ckdgen.imbi.uni-freiburg.de).

## Discussion

The eMERGE consortium has pioneered standardized electronic phenotyping algorithms based on EHR data^37^. Such electronic phenotypes can be used to efficiently identify and recruit patients into cohort studies and pragmatic clinical trials^38,39^ and for large-scale population health research or precision medicine studies^40–43^. Additional uses include determining clinical outcomes^44^, identifying novel genotype-phenotype associations^43^, and implementing clinical decision support systems within EHRs^38,45^.

CKD is generally underdiagnosed and represents a growing public health problem worldwide^20^. Because its diagnosis relies almost entirely on blood and urine tests, CKD is ideal for developing a computable EHR-based definition. Our proposed modular CKD algorithm’s unique feature is that it performs an automated diagnosis and staging across the entire KDIGO grid, allowing for risk stratification at a higher resolution than the previously proposed simpler phenotyping methods^46,47^. Moreover, in our analyses, we demonstrate that our electronic phenotype is accurate, portable, and scalable to large EHR datasets involving over a million of individuals. In addition to extensive manual validations, we provide evidence for genetic validity by in silico replication of known genetic associations for CKD.

Although conceptually simple, our algorithm overcomes several important practical challenges stemming from real-life limitations of EHR data. Any CKD diagnostic algorithm based on serum Cr measurements needs to overcome potential misclassification due to physiologic (e.g. volume depletion) or disease-related (e.g. acute kidney injury) fluctuations of single time point serum Cr values. Our algorithm includes a judicious criterion for chronicity, requiring CKD duration for over 90 days as documented by repeat blood or urine tests, or documentation by a prior diagnostic code. We also carefully define qualifying eGFR as the one that does not co-occur with acute kidney injury, volume depletion, or critical illness.

One of the greatest obstacles for developing our algorithm was the fact that the A-staging requires accurate estimation of daily urine albumin excretion. Estimating albuminuria using EHR data is not straightforward, mainly because an array of urine protein tests is used in clinical practice. Current guidelines recommend spot UACR as an optimal method to quantify albuminuria. However, recent studies using EHR have shown that even patients at high risk for CKD frequently receive only DSP tests^48,49^. We used a supervised machine-learning approach to design accurate classifiers translating the most commonly used urinalysis tests to the KDIGO-defined A-stages. We also demonstrated that our urinalysis-based A-stage classifier outperforms the alternative methods published while our study was under review^24^. This improved performance is most likely due to the fact that our classifiers include urine specific gravity in addition to DSP, reducing misclassification due to variations in urine concentration. Our convenient classifiers are fully automated and can be used to improve phenotype definitions in large scale genetic, epidemiologic, and interventional studies of CKD.

Several limitations of our approach should be noted. First, the successful detection and staging of CKD using our approach is dependent on the availability of relevant blood and urine tests in medical records. Because serum Cr is usually determined as part of routine health screening tests, most patients have serum Cr available for G-staging. However, urine tests are performed less frequently, and the A-staging could therefore be incomplete due to missing data. Second, our algorithm performs G-staging using the CKD-EPI formula for GFR estimation in adults^50^. The CKD-EPI formula utilizes age, sex, and race in addition to serum Cr to determine eGFR. The race information is problematic, since it is based on self-report and this information is frequently inaccurate in medical records^51^. Additionally, Cr-based GFR estimation in individuals of diverse or admixed ancestries may be inaccurate, since CKD-EPI was derived on a cohort composed of only White and Black Americans. Although CKD-EPI equation presently provides the most accurate means for GFR estimation, it could be easily replaced by any future equations that replace the race variable without other major changes to the algorithm^52^. Similarly, estimation of GFR in pediatric patients may be less accurate compared to adults, but the formula used by the algorithm could be updated once more accurate equations become available. Third, our algorithm is currently designed for detection and staging of all cause CKD along the two axes of A-by-G grid. Adding the third axis of CKD subtype (i.e. primary disease subtype) could substantially enhance our phenotyping. However, automated determination of primary kidney diagnoses using medical records proves to be challenging for a number of reasons, including large etiologic heterogeneity, long time period of CKD progression that may not be well covered by EHRs, inadequate classification of kidney disease subtypes by older billing codes (e.g. ICD-9-CM), and the fact that a kidney biopsy (the gold standard for primary kidney disease diagnosis) is underutilized in clinical practice^53^. As a result, the primary cause of kidney disease is often difficult to establish with certainty, even by manual review of medical records.

Despite these limitations, we demonstrate that our electronic phenotype provides effective means for clinical detection and staging of CKD. There are several ways in which automated diagnosis of CKD could substantially enhance clinical care. First, algorithmic diagnosis could enhance physician and patient awareness of the disease. Recent studies show that less than 10% of patients with early CKD (stages 1–3), and only half (52%) of those with severe CKD (stage 4) are aware of having a kidney problem^11^. Given that CKD progression is often irreversible and early interventions are considered to be most effective, our algorithm could address this issue by flagging CKD diagnoses in medical records, alerting both clinicians and patients. The clinical benefit may be greatest for the detection of mild (G1A2-3) disease that may benefit most from early therapeutic interventions, such as renin-angiotensin system blockade^15^ or the use of sodium-glucose cotransporter-2 inhibitors^16^.

We recommend a confirmation of A-staging by UACR, which represents the gold standard underutilized in clinical screening for CKD^54^. Once confirmed, additional tests may be needed to define the cause of renal damage, including renal imaging, diabetes screening, blood pressure monitoring, and possibly a renal biopsy. Second, our algorithm also enables the implementation of stage-specific recommendations for the management of common complications of more advanced CKD. For example, the management of CKD-associated anemia and renal osteodystrophy are complex, stage-specific, and expensive^55^. Our staging algorithm could be easily incorporated into a clinical decision support system that helps treating physicians to achieve target levels of hemoglobin or parathyroid hormone by suggesting appropriate stage-specific use of oral and injectable medications^56^.

To assure the algorithm’s interoperability and portability, we used a parameterized and modularized implementation which can be easily customized to different data models (e.g. i2b2, OMOP), commonly used data elements available in different EHR systems (person id, event type, event time), and data dictionaries which are fully compatible with commonly used coding schemes (e.g. ICD-9-CM, ICD-10-CM, SNOMED for diagnosis). We provide open-source software on PheKB website (https://phekb.org/phenotype/chronic-kidney-disease) for a straightforward local implementation of the CKD phenotype, indicating parts of the code that require local customization. This code can be used as a starting point for building CKD alert systems or related clinical decision support applications.

In addition to clinical utility, we demonstrate that our algorithm has multiple research applications, from observational inference to genetics and any other population health research based on EHR, as demonstrated by our large-scale comorbidity, heritability, and GWAS analyses. Our GWAS involving 25,377 Europeans recapitulates genome-wide significant CKD associations at the *UMOD* locus originally discovered at comparable significance in the analysis of 19,877 Europeans ascertained using traditional methods^57^. This suggests that our algorithm’s applications to large biobanks with genetic data linked to medical records could empower new genetic discoveries for CKD.

The studies using our electronic phenotype have already provided valuable insights into the genetic architecture of CKD. Our pedigree-based analyses support a substantial hereditary component to CKD and highlight ancestral disparities in genetic susceptibility to kidney diseases. Despite high heritability in pedigree-based analyses, the SNP-based heritability of CKD was estimated at only ~1% based on the largest available studies performed predominantly in European-ancestry cohorts. Although SNP-based heritability of eGFR is higher (estimated at up to 8%) compared to CKD, this estimate is more likely to be confounded by inherited differences in muscle mass, Cr production, and Cr metabolism in population-based studies, and thus may be less reflective of the true heritability of CKD as defined by reduced Cr clearance. The low estimates of SNP-based heritability of CKD (and their relatively wide 95% confidence intervals) are likely due to the etiologic heterogeneity of CKD, and the fact that the existing GWAS for CKD are still of limited sample size. Widespread application of our algorithm to big biobanks should empower larger GWAS for CKD, providing more accurate estimates.

The wide gap between pedigree-based and SNP-based heritability is not unique to CKD, and has been reported for other complex traits^58^. There are several potential explanations for this observation. First, the estimates of pedigree-based heritability could be partially inflated by shared environment and epigenetic effects. Although we make an attempt to control for shared household in our heritability estimates, environmental effects are generally difficult to adjust for in family-based studies. Second, the heritability gap may be due to additive modeling not accounting for non-additive SNP effects, such as recessive, dominant, gene-gene, or gene-environment interaction effects. For example, *APOL1* risk genotype effects are contributing to family-based heritability in African Americans, but are not captured by SNP-based heritability, because the genetic risk model is recessive and *APOL1* locus is missed in GWAS dominated by Europeans. Third, the heritability gap could be explained by rare Mendelian or structural variants that are not accounted for in the estimation of SNP-based heritability. This may indeed represent the most likely explanation given that recent exome sequencing studies in CKD demonstrate that up to 1 in 10 adult cases may be attributable to a monogenic disease variant^59^. Moreover, up to 7% of pediatric CKD and up to 6% of congenital kidney defects could be attributable to genomic disorders^60–62^.

Taken together, the applications of our electronic phenotype to GWAS and pedigree-based analysis provide support for a strong genetic predisposition to CKD, but low SNP-based heritability. Our findings are consistent with the notion that CKD may not represent a single phenotype but rather a collection of genetically and phenotypically heterogeneous diseases encompassing multiple Mendelian subtypes, as well as disorders of more complex genetic determination. These observations have important implications for the implementation of kidney precision medicine. For example, the approaches that combine diagnostic sequencing with polygenic risk scores for specific subtypes of kidney diseases may be better suited for clinical risk stratification compared to polygenic risk scores based on GWAS for eGFR alone^63^. Future improvements of e-phenotyping for CKD are likely to involve automated CKD subtype determination, and more accurate methods for estimation of GFR in adult and pediatric patients of diverse ancestral backgrounds.

## Methods

### Algorithm development

We used (1) the NKF’s KDOQI guidelines^19^, (2) the KDIGO Clinical Practice Guideline for the Evaluation and Management of CKD^9,10^, and (3) domain expert knowledge in nephrology to define CKD cases and controls using lab measurements in combination with diagnosis and procedure codes (Fig. 1). Subjects who required kidney transplant or dialysis were defined as having reached ESRD. To define CKD in subjects with a native kidney function, we used (1) the most recent eGFR, (2) the eGFR measured at least 3 months before the most recent eGFR, (3) CKD and relevant kidney disease diagnosis codes, and (4) any of the five commonly used urine tests that detect albuminuria or proteinuria, including semi-quantitative urine dipstick tests. To accomplish the G-staging, we designed a “G-Stage Classifier” that uses the most recent eGFR. To distinguish CKD from the abnormal kidney function that is caused by acute kidney injury or other acute physiological states. Any eGFR measures that co-occur with such conditions within 1-month, including value(s) measured during a period of critical illness were excluded. The A-staging was performed using an “A-Stage Classifier” based on the most recent urine protein or albumin test. We define subjects with normal renal function and no albuminuria (G1A1 controls) as individuals whose most recent eGFR is within normal range, and who lack any diagnostic or procedure codes related to CKD and have no evidence of albuminuria on most recent urine test. We define subjects with normal renal function (G1 controls) as individuals whose most recent eGFR is within normal range and who lack any diagnostic or procedure codes related to CKD, but who have no available urine tests precluding the determination of A-stage.

### G-Stage Classifier

We use most recent eGFR values to perform G-staging. The eGFR is estimated using CKD-EPI formula in adults^50^ and Bedside Schwartz equation in pediatric patients (age < 18 years old)^64,65^. Since the Bedside Schwartz equation requires height concurrent with serum Cr, and the height data do not always coincide with Cr data, we used a simple height extrapolation method (Eq. 1). The precise G-stage is determined using simple threshold-based rules, as depicted in Fig. 1b.

1 $$Ht = Ht{\,}_{pre} + \frac{{(Ht{\,}_{pre} - Ht{\,}_{post})(HtDateInDays{\,}_{pre} - HtDateInDays)}}{{HtDateInDays{\,}_{pre} - HtDateInDays{\,}_{post}}}$$

### A-Stage Classifier

In order to utilize all of the available urine tests for A-staging, we leveraged “real life” EHR data and implemented a simple machine-learning-based approach based on ordinal regression. Our method aimed to harmonize commonly used urine tests that quantify proteinuria to predict albuminuria stage for each patient (Fig. 1c).

We considered the following predictors of A-stage: Urine Albumin-to-Cr-Ratio (UACR, guideline-recommended gold standard), 24-h urine collection for albumin (A24), Urine Protein-to-Cr-Ratio (UPCR), 24-h urine collection for protein (P24) and urine dipstick protein test (DSP). For the purpose of our algorithm, we assume that P24 [mg/24 h] and UPCR [mg/g Cr] are numerically equivalent. While A-stages can be derived directly from UACR and A24 using KDIGO-recommended cut-offs, our algorithm aimed to perform A-staging using UPCR, P24, or DSP. For this purpose, we designed two separate supervised machine-learning approaches.

### UPCR-based A-Stage classification

The first approach aimed to build an ordinal classifier which maps UPCR or P24 values to individual A-stages. For our training set, we identified all same day paired urine tests for UPCR and UACR within the Columbia EHR (*n* = 4641 paired measurements). We used UACR as the gold standard to define the A-stage (A1, A2, and A3). Using this training set, we next applied an ordinal regression-based approach to construct the A-stage classifier. Feature selection was performed with a goal to minimize mean squatted error of the model. The features tested included log-transformed UPCR, age, sex, diabetes, race, ethnicity. For each model, we estimated model coefficients, used them to compute probabilities of A1, A2, and A3, and maximized over these probabilities to predict the most likely stage for any given set of predictor values. We then used a 10-fold cross-validation approach, enabling calculation of mean squared error (MSE) for our ordinal classifier (Eq. 2), as well as accuracy, sensitivity, and specificity with their 95% confidence intervals. In this analysis, log-transformed UPCR alone represented the strongest predictor of A-stage, and the addition of age, sex, diabetes, race, or ethnicity provided no additional improvements in model performance.

2 $$MSE = \frac{{\mathop {\sum}\nolimits_{i = 1}^{10} {error\;rate} }}{{10}} \pm 1.96 \times \frac{{sd(error\;rate)}}{{sqrt(10)}}$$

To validate our model, we tested two external datasets of paired UPCR-UACR measurements from medical records of the UMN (*n* = 8688) and VU (*n* = 5770). The MSE, accuracy, sensitivity, and specificity metrics were remarkably similar between internal and external validation datasets, thus we build a final predictive model that was derived from the entire dataset of 19,099 paired observations across all three institutions (Supplementary Tables 1–2). This final model was used in our algorithm. This classifier had 86.7%, 80.0%, and 92.3% accuracy for A1, A2, and A3, respectively. The specificity was high for all A-stages (87.1–94.8%), but the sensitivity was lower for A2 (63.5%) compared to A1 and A3 (86.2–86.7%). This is consistent with the UPCR method being less accurate at a lower level of albuminuria.

In the second approach, we built an A-stage predictor using DSP from routine urinalyses. There are two major challenges that we aimed to address. First, the semi-quantitative DSP grade is dependent on the concentration of urine, which is affected by a number of confounding factors, such as fluid intake, volume status, or use of diuretics. Second, different institutions use different semi-quantitative scales to report DSP grade. To address the first problem, we again used a supervised machine-learning approach, but now we incorporated urine specific gravity (SG) measured at the same time as DSP as an additional input feature. For the second problem, we identified two most common urinalysis scales and developed an A-stage classifier using two separate training sets for these scales: Scale 1 (negative, trace, 1+, 2+, 3+, 4+) and Scale 2 (negative, trace, 10, 30, 100, 300, >=300).

To develop a Scale 1-based classifier, we used 12,185 simultaneous DSP and UACR measurements from the CUIMC EHR system (Supplementary Table 3). The final Scale 1 classifier had the accuracy of 80.9%, 76.0%, and 94.3% for A1, A2, and A3, respectively, by 10-fold cross-validation. For Scale 2-based classifiers we used a similar dataset of 35,891 paired measurements identified within the UMN and additional 7595 paired DSP-UACR measurements from VU (Supplementary Table 4). Each classifier was built using ordinal regression with DSP, SG, age and sex as independent predictors of the A-stage. For each training dataset, we used 10-fold cross-validation approach, derived MSE, accuracy, sensitivity, and specificity with 95% confidence intervals. Similar to our first approach, neither age nor sex increased our predictive ability and these predictors were subsequently excluded from the model. However, the addition of urine specific gravity to DSP grade significantly improved the model performance regardless of the scale. We also explored more complex machine-learning methods and several other features, including diabetes, race, ethnicity, and other urinalysis variables, such as urine glucose, ketones, pH, blood, leukocyte esterase, nitrates, and bilirubin, however, none of these complex models outperformed a simple ordinal classifier based on combined DSP and SG.

Because the models based on UMN and VU datasets had comparable performance in cross-validation, we decided to pool these datasets (*n* = 43,486 paired measurements) to derive the final classifier. The performance of the Stage 2-classifier was tested by 10-fold cross-validation (Supplementary Table 5). The final Scale 2 classifier had the accuracy of 82.2%, 78.5%, and 95.3% for A1, A2, and A3, respectively, by 10-fold cross-validation. The summary of predicted probabilities of A-stages across a range of individual predictors are illustrated in Supplementary Fig. 1 for all three (UPCR, DSP1, and DSP2) final classifiers.

While this work was under review, alternative methods of UACR estimation from UPCR and DSP have been proposed by the CKD Prognosis Consortium^24^. Therefore, we performed additional tests of our ordinal classifiers with performance comparisons to the newly proposed crude and adjusted linear regression-based models. In two independent datasets (13,134 paired UPCR-UACR measurements and 6695 paired UA-UACR measurements) we demonstrated that the performance of our A-stage classifiers was consistent between the testing and discovery datasets, and generally comparable to the newly published methods. Similar to our study, the CKD Prognosis Consortium models additionally adjusted for sex, diabetes, and hypertension did not perform better over simple models based on UPCR or DSP alone (Supplementary Table 6).

### Algorithm implementation

To enable portable implementation, a parameterized and modularized algorithm query was developed^66^. This query template has two major features. First, complex query logic is built from several simple query block modules, each serving a single function. In the modularized query, the first query block is to retrieve all phenotype-related variables from the source data and store them in a temporary table for later query blocks use. This way, the data retrieval and algorithm logic parts can be separated. This logical separation has been explored by the Arden syntax, which is designed to share task-specific knowledge implementations across institutions^67^. Another feature of our query template is to encapsulate source database schema and coding dictionary into parameters, which can be replaced at the execution. Both features make the query template easily adaptable to different data environments. To ensure compatible implementation across sites, we also use national standard terminologies to define diagnosis, procedure and laboratory tests. We define diagnosis codes using ICD-9-CM and ICD-10-CM, procedure codes using CPT-4, ICD-9-PCS, and ICD-10-PCS. For laboratory tests, we identified all relevant LOINC codes. Since different institutions may use local coding for laboratory tests, institution-specific coding review is required before implementation at each site. The algorithm and all associated data dictionaries have been deposited in the public Phenotype Knowledge Base^25^ (https://phekb.org/phenotype/chronic-kidney-disease).

### Algorithm validations

We determined the PPV of the algorithm by conducting 451 blinded manual chart reviews as a gold standard across three large US institutions. For internal validation at CUIMC, we selected 251 charts (189 CKD cases evenly distributed across all disease stages and 62 healthy non-CKD controls) with adequate data within the EHR. Two blinded nephrologists were asked to make the CKD diagnosis and stage the disease based on the latest lab values and clinical chart data; a third expert blinded to the algorithm results resolved any discrepancies. For external validation, we performed manual review of additional 200 charts (160 CKD cases evenly distributed across all disease stages and 40 healthy non-CKD controls) within the VU and Mayo Clinic EHR system. We calculated overall PPVs as well as PPVs by case/control status, by institution, and by CKD stage (Table 2).

For secondary validation, and to calculate diagnostic sensitivity and specificity, we used an independent case-control dataset consisting of 1136 cases (defined as patients with an outpatient visit to the Columbia CKD clinic and carrying at least one ICD code consistent with CKD as determined by a nephrologist) and 1214 controls (defined as women attending a prenatal screening visit at Columbia during the same time period that do not have any billing code consistent with CKD in their medical record). The algorithm had specificity of 97%, sensitivity of 87%, PPV of 97%, NPV of 89%, and F1 measure of 92% for discriminating CKD patients from healthy controls (Supplementary Table 7).

### CKD comorbidities

We applied our algorithm to the entire Columbia CDW covering data from 1997 to 2017. Among 1,365,098 patients with at least one serum creatinine value available, the algorithm had sufficient data to stage 672,858 individuals. We used the AHRQ Elixhauser Comorbidity Index that defines 40 comorbidity measures from ICD-9-CM and ICD-10-CM codes for comorbidity analysis^26,27^. The prevalence of CKD by A and G stage, along with the prevalence of related comorbidities were calculated and adjusted for age and sex using U.S. 2000 Standard Population (https://seer.cancer.gov/stdpopulations). The association screen for CKD comorbidities was performed by evaluating co-occurrence of CKD with all other diagnostic and procedure codes by A and G stage. We tested for significant additive patterns in age and sex-adjusted comorbidities across the A-by-G grid using logistic regression; each comorbidity was used as an outcome, and A and G stages were used as ordinal predictors with age and sex as covariates in the model. Using these models, we tested for an independent additive effect of A and G stages on each comorbid condition using Wald test. Given a total of 40 independent comorbidities tested with two tests per each comorbidity, we used a Bonferroni-adjusted alpha of 0.05/80 = 6.25 × 10^−4^ to declare statistical significance.

### Observational heritability

We used the RIFTEHR algorithm^22^ to infer familial relationships among individuals with inpatient EHR records at CUIMC. Briefly, a total of 3,244,380 unique relationships have been identified at Columbia based on emergency contact information combined with relationship inference as described previously^22^. We grouped individuals into families by identifying disconnected relationship sub-graphs and found 223,307 families ranging from 2 to 134 members per family. We next intersected the pedigree dataset with the output of the CKD algorithm applied to the CUIMC EHR. This allowed us to estimate observational heritability for our electronic CKD phenotypes, including eGFR, any albuminuria (A2 or A3), heavy albuminuria (A3), the diagnosis of any CKD, moderate CKD (stage 3 or greater), and advanced CKD (stage 4 or greater). We modeled heritability under additive genetic model with phenotype adjusted for age, sex, race/ethnicity, and common environment (approximated by a term that used the mother ID as the household ID). We used SOLAR*Strap*, a repeated subsampling procedure in which each subsampled set of families is used to estimate heritability using SOLAR^30^. These estimates are then averaged to produce a robust heritability estimate that is less prone to ascertainment bias^22^.

### Genome-wide association studies

For the purpose of genetic studies, we implemented the CKD phenotype across the entire eMERGE-III network. The network provides access to EHR information linked to GWAS data for 105,108 individuals. Detailed pre-imputation quality control pipelines for genetic data of the eMERGE-III consortium have previously been described^21^. Briefly, GWAS datasets were imputed using the latest multiethnic Haplotype Reference Consortium (HRC) panel. The imputation was performed in 81 individual batches across the 12 contributing medical centers participating in eMERGE-I, II, and III. For post-imputation analyses, we included only markers with MAF ≥ 0.01 and *R*^2^ ≥ 0.8 in ≥75% of batches. These quality control analyses were performed using a combination of VCFtools, PLINK, and custom scripts in PYTHON and R^68–70^. To assess population stratification and remove population outliers, we applied a principal component analysis using FlashPCA^71^. We applied k-means clustering algorithm to the PCA data to split the overall cohort into the three major ancestral clusters based on similarity to reference populations from the 1000 Genomes Project (European, African and East Asian). All genome-wide association analyses were subsequently performed within each major ancestral group, after adjustment for age, sex, site, and significant principal components re-derived for each ancestral cluster (the significance of principal components was determined using the Tracy–Widom test). Each site participating in eMERGE-III implemented our electronic phenotype and provided the algorithm output for linkage with the genetic data. The association analyses of binary traits (CKD vs. control) were performed using logistic regression. We used a dosage method under additive genotype coding to account for imputation uncertainty. For each SNP, we derived pooled effect estimates, their standard errors, and 95% confidence intervals. Genome-wide distributions of *P* values were examined visually using quantile-quantile plots and we estimated genomic inflation factors for each genome-wide scan^72^. We used the generally accepted alpha = 5 × 10^−8^ to declare genome-wide significance^73^. To estimate the fraction of additive genetic variance contributed by genome-wide SNP data and to derive pairwise genetic correlations between phenotypes, we used the linkage disequilibrium score regression (LDSC) method^74^.

### Phenome-wide association studies

We used the latest release of eMERGE-III data for PheWAS. The phenotype data consisted of 19,853 distinct ICD-9-CM codes for 105,108 individuals with genotype data. The ICD-9-CM codes were mapped to phecodes and PheWAS was performed using the PheWAS R package^23^. The package uses pre-defined “control” groups for each phecode “case” grouping. In total 1804 phecodes were tested using age, sex, center, and principal component-adjusted logistic regression model with each phecode case-control status as an outcome. The genotype predictors were coded under additive model for risk alleles. We set the Bonferroni-corrected statistical significance threshold at 2 × 10^−5^ (0.05/1804) to control for the number of phecodes tested.

### Ethics

The study was approved by the Columbia University Institutional Review Board (IRB protocol numbers IRB-AAAP7926 and IRB-AAAO4154) and individual IRBs at all eMERGE-III network sites contributing human genetic and clinical data. Our large scale heritability and comorbidity analyses based on the Columbia Data Warehouse were performed under an approved waiver of consent. BioVU operated on an opt-out basis until January 2015 and on an opt-in basis since. The phenotypic data in BioVU are all de-identified and the study was designated “non-human subjects” research by the Vanderbilt Institutional Review Board. All other eMERGE participants provided informed consent to participate in genetic studies.

### Reporting summary

Further information on experimental design is available in the Nature Research Reporting Summary linked to this paper.

## Supplementary information

Reporting Summary

Supplementary Information

Supplementary Data 1

Supplementary Data 2

## Data Availability

The software and documentation of the Electronic CKD Phenotype can be found on the Phenotype Knowledge Database (PheKB) website (https://phekb.org/phenotype/chronic-kidney-disease). The PheKB documentation also includes a detailed list of all ICD-9-CM, ICD-10-CM, SNOMED, lab LOINC and procedure CPT codes used by the algorithm. The eMERGE-III genetic datasets with linked phenotypes are accessible through dbGAP (accession number: phs001584.v1.p1).

## References

[1] Centers for Disease Control and Prevention. Chronic Kidney Disease (CKD) Surveillance Project website. https://nccd.cdc.gov/CKD.

[2] Bowe B (2018). Changes in the US Burden of Chronic Kidney Disease From 2002 to 2016: An Analysis of the Global Burden of Disease Study. JAMA Netw. Open.

[3] Collaboration, G.B.D.C.K.D. (2020). Global, regional, and national burden of chronic kidney disease, 1990-2017: a systematic analysis for the Global Burden of Disease Study 2017. Lancet.

[4] United States Renal Data System (USRDS) 2018 Annual Data Report. www.usrds.org.

[5] Genovese G (2010). Association of trypanolytic ApoL1 variants with kidney disease in African Americans. Science.

[6] Parsa A (2013). APOL1 risk variants, race, and progression of chronic kidney disease. N. Engl. J. Med..

[7] Köttgen A (2010). New loci associated with kidney function and chronic kidney disease. Nat. Genet..

[8] Wuttke M (2019). A catalog of genetic loci associated with kidney function from analyses of a million individuals. Nat. Genet.

[9] Stevens PE, Levin A (2013). & Kidney Disease: Improving Global Outcomes Chronic Kidney Disease Guideline Development Work Group, M. Evaluation and management of chronic kidney disease: synopsis of the kidney disease: improving global outcomes 2012 clinical practice guideline. Ann. Intern Med.

[10] Kidney Disease: Improving Global Outcomes (KDIGO) CKD Work Group. KDIGO 2012 Clinical Practice Guideline for the Evaluation and Management of Chronic Kidney Disease. *Kidney Int. Suppl.* 1–150 (2013).

[11] Dharmarajan SH (2017). State-level awareness of chronic kidney disease in the U.S. Am. J. Prev. Med.

[12] Cholesterol Treatment Trialists, C. (2016). Impact of renal function on the effects of LDL cholesterol lowering with statin-based regimens: a meta-analysis of individual participant data from 28 randomised trials. Lancet Diabetes Endocrinol..

[13] Group SR (2015). A randomized trial of intensive versus standard blood-pressure control. N. Engl. J. Med.

[14] Coca SG, Ismail-Beigi F, Haq N, Krumholz HM, Parikh CR (2012). Role of intensive glucose control in development of renal end points in type 2 diabetes mellitus: systematic review and meta-analysis intensive glucose control in type 2 diabetes. Arch. Intern Med.

[15] Xie X (2016). Renin-angiotensin system inhibitors and kidney and cardiovascular outcomes in patients with CKD: a Bayesian network meta-analysis of randomized clinical trials. Am. J. Kidney Dis..

[16] Heerspink HJL (2020). Dapagliflozin in patients with chronic kidney disease. N. Engl. J. Med.

[17] Perkovic V (2019). Canagliflozin and renal outcomes in type 2 diabetes and nephropathy. N. Engl. J. Med.

[18] Go AS, Chertow GM, Fan D, McCulloch CE, Hsu C-y (2004). Chronic kidney disease and the risks of death, cardiovascular events, and hospitalization. N. Engl. J. Med..

[19] Levey AS (2003). National Kidney Foundation practice guidelines for chronic kidney disease: evaluation, classification, and stratification. Ann. Intern Med.

[20] Shlipak MG (2021). The case for early identification and intervention of chronic kidney disease: conclusions from a Kidney Disease: Improving Global Outcomes (KDIGO) Controversies Conference. Kidney Int.

[21] Stanaway IB (2019). The eMERGE genotype set of 83,717 subjects imputed to ~40 million variants genome wide and association with the herpes zoster medical record phenotype. Genet Epidemiol..

[22] Polubriaginof FCG (2018). Disease heritability inferred from familial relationships reported in medical records. Cell.

[23] Carroll RJ, Bastarache L, Denny JC (2014). R PheWAS: data analysis and plotting tools for phenome-wide association studies in the R environment. Bioinformatics.

[24] Sumida K (2020). Conversion of urine protein-creatinine ratio or urine dipstick protein to urine albumin-creatinine ratio for use in chronic kidney disease screening and prognosis: an individual participant-based meta-analysis. Ann. Intern Med..

[25] Kirby JC (2016). PheKB: a catalog and workflow for creating electronic phenotype algorithms for transportability. J. Am. Med Inf. Assoc..

[26] Moore BJ, White S, Washington R, Coenen N, Elixhauser A (2017). Identifying increased risk of readmission and in-hospital mortality using hospital administrative data: the AHRQ Elixhauser Comorbidity Index. Med Care.

[27] Elixhauser A, Steiner C, Harris DR, Coffey RM (1998). Comorbidity measures for use with administrative data. Med. Care.

[28] Marwick TH (2019). Chronic kidney disease and valvular heart disease: conclusions from a Kidney Disease: Improving Global Outcomes (KDIGO) Controversies Conference. Kidney Int.

[29] McQuillan R, Jassal SV (2010). Neuropsychiatric complications of chronic kidney disease. Nat. Rev. Nephrol..

[30] Almasy L, Blangero J (1998). Multipoint quantitative-trait linkage analysis in general pedigrees. Am. J. Hum. Genet.

[31] Fox CS (2004). Genomewide linkage analysis to serum creatinine, GFR, and creatinine clearance in a community-based population: the Framingham Heart Study. J. Am. Soc. Nephrol..

[32] Langefeld CD (2004). Heritability of GFR and albuminuria in Caucasians with type 2 diabetes mellitus. Am. J. Kidney Dis..

[33] Bochud M (2005). Heritability of renal function in hypertensive families of African descent in the Seychelles (Indian Ocean). Kidney Int.

[34] Mottl AK (2008). Linkage analysis of glomerular filtration rate in American Indians. Kidney Int..

[35] Hunt SC (2002). Linkage of creatinine clearance to chromosome 10 in Utah pedigrees replicates a locus for end-stage renal disease in humans and renal failure in the fawn-hooded rat. Kidney Int..

[36] Pattaro C (2016). Genetic associations at 53 loci highlight cell types and biological pathways relevant for kidney function. Nat. Commun..

[37] Newton KM (2013). Validation of electronic medical record-based phenotyping algorithms: results and lessons learned from the eMERGE network. J. Am. Med. Inform. Assoc..

[38] Richesson RL (2013). Electronic health records based phenotyping in next-generation clinical trials: a perspective from the NIH Health Care Systems Collaboratory. J. Am. Med. Inform. Assoc..

[39] Casey JA, Schwartz BS, Stewart WF, Adler NE (2016). Using electronic health records for population health research: a review of methods and applications. Annu Rev. Public Health.

[40] Wei WQ, Denny JC (2015). Extracting research-quality phenotypes from electronic health records to support precision medicine. Genome Med..

[41] Hripcsak G (2019). Facilitating phenotype transfer using a common data model. J. Biomed. Inf..

[42] Rasmussen LV (2014). Design patterns for the development of electronic health record-driven phenotype extraction algorithms. J. Biomed. Inf..

[43] Kho AN (2011). Electronic Medical Records for Genetic Research: Results of the eMERGE Consortium. Sci. Transl. Med..

[44] Robb MA (2012). The US Food and Drug Administration’s Sentinel Initiative: Expanding the horizons of medical product safety. Pharmacoepidemiol. Drug Saf..

[45] Jensen PB, Jensen LJ, Brunak S (2012). Mining electronic health records: towards better research applications and clinical care. Nat. Rev. Genet.

[46] Norton JM (2019). Development and validation of a pragmatic electronic phenotype for CKD. Clin. J. Am. Soc. Nephrol..

[47] Nadkarni GN (2014). Development and validation of an electronic phenotyping algorithm for chronic kidney disease. AMIA Annu Symp. Proc..

[48] Covic AMC (2001). A family-based strategy to identify genes for diabetic nephropathy. Am. J. kidney Dis..

[49] Iyengar SK (2003). Linkage analysis of candidate loci for end-stage renal disease due to diabetic nephropathy. J. Am. Soc. Nephrol..

[50] Levey AS (2009). A new equation to estimate glomerular filtration rate. Ann. Intern Med.

[51] Polubriaginof FCG (2019). Challenges with quality of race and ethnicity data in observational databases. J. Am. Med Inf. Assoc..

[52] Levey AS, Titan SM, Powe NR, Coresh J, Inker LA (2020). Kidney Disease, Race, and GFR Estimation. Clin. J. Am. Soc. Nephrol..

[53] Poggio ED (2020). Systematic review and meta-analysis of native kidney biopsy complications. Clin. J. Am. Soc. Nephrol..

[54] Fisher H, Hsu CY, Vittinghoff E, Lin F, Bansal N (2013). Comparison of associations of urine protein-creatinine ratio versus albumin-creatinine ratio with complications of CKD: a cross-sectional analysis. Am. J. Kidney Dis..

[55] Kidney Disease Improving Global Outcomes (KDIGO). Chapter 3: Management of progression and complications of CKD. *Kidney Int. Suppl.* 3, 73–90 (2013). 10.1038/kisup.2012.66 PMC4283680 25598999

[56] Patwardhan MB, Kawamoto K, Lobach D, Patel UD, Matchar DB (2009). Recommendations for a clinical decision support for the management of individuals with chronic kidney disease. Clin. J. Am. Soc. Nephrol..

[57] Kottgen A (2009). Multiple loci associated with indices of renal function and chronic kidney disease. Nat. Genet.

[58] Manolio TA (2009). Finding the missing heritability of complex diseases. Nature.

[59] Groopman EE (2019). Diagnostic utility of exome sequencing for kidney disease. N. Engl. J. Med.

[60] Verbitsky M (2015). Genomic imbalances in pediatric patients with chronic kidney disease. J. Clin. Investig..

[61] Sanna-Cherchi S (2012). Copy-number disorders are a common cause of congenital kidney malformations. Am. J. Hum. Genet.

[62] Verbitsky M (2019). The copy number variation landscape of congenital anomalies of the kidney and urinary tract. Nat. Genet.

[63] Liu L, Kiryluk K (2018). Genome-wide polygenic risk predictors for kidney disease. Nat. Rev. Nephrol..

[64] Schwartz GJ (2009). New equations to estimate GFR in children with CKD. J. Am. Soc. Nephrol..

[65] Schwartz GJ, Work DF (2009). Measurement and estimation of GFR in children and adolescents. Clin. J. Am. Soc. Nephrol..

[66] Shang, N., Weng, C. & Hripcsak, G. A method for enhancing the portability of electronic phenotyping algorithms: An eMERGE Pilot Study. in AMIA 2016 Annual Symposium (Chicago, 2016).

[67] Hripcsak G, Ludemann P, Pryor TA, Wigertz OB, Clayton PD (1994). Rationale for the Arden Syntax. Computers Biomed. Res..

[68] Purcell S (2007). PLINK: a tool set for whole-genome association and population-based linkage analyses. Am. J. Hum. Genet.

[69] Danecek P (2011). The variant call format and VCFtools. Bioinformatics.

[70] Denny JC (2010). PheWAS: demonstrating the feasibility of a phenome-wide scan to discover gene-disease associations. Bioinformatics.

[71] Abraham G, Inouye M (2014). Fast principal component analysis of large-scale genome-wide data. PLoS One.

[72] Devlin B, Roeder K, Bacanu SA (2001). Unbiased methods for population-based association studies. Genet Epidemiol..

[73] Dudbridge F, Gusnanto A (2008). Estimation of significance thresholds for genomewide association scans. Genet Epidemiol..

[74] Bulik-Sullivan BK (2015). LD Score regression distinguishes confounding from polygenicity in genome-wide association studies. Nat. Genet.

