# Supplementary material for: Medical records-based chronic kidney disease phenotype for clinical care and “big data” observational and genetic studies
Source: NPJ Digit Med. 2021 Apr 13;4:70. doi: 10.1038/s41746-021-00428-1 (PMC8044136; doi:10.1038/s41746-021-00428-1)
Supplement: Supplementary file 1 — Reporting Summary [file 41746_2021_428_MOESM1_ESM.pdf]

nature research

Corresponding author(s): Krzysztof Kiryluk

Last updated by author(s): Jan 12, 2021

Reporting Summary

Nature Research wishes to improve the reproducibility of the work that we publish. This form provides structure for consistency and transparency in reporting. For further information on Nature Research policies, see our Editorial Policies and the Editorial Policy Checklist.

Statistics

For all statistical analyses, confirm that the following items are present in the figure legend, table legend, main text, or Methods section.

n/a	Confirmed	
☐	☒	The exact sample size (*n*) for each experimental group/condition, given as a discrete number and unit of measurement
☐	☒	A statement on whether measurements were taken from distinct samples or whether the same sample was measured repeatedly
☐	☒	The statistical test(s) used AND whether they are one- or two-sided<br>*Only common tests should be described solely by name; describe more complex techniques in the Methods section.*
☐	☒	A description of all covariates tested
☐	☒	A description of any assumptions or corrections, such as tests of normality and adjustment for multiple comparisons
☐	☒	A full description of the statistical parameters including central tendency (e.g. means) or other basic estimates (e.g. regression coefficient) AND variation (e.g. standard deviation) or associated estimates of uncertainty (e.g. confidence intervals)
☐	☒	For null hypothesis testing, the test statistic (e.g. *F*, *t*, *r*) with confidence intervals, effect sizes, degrees of freedom and *P* value noted<br>*Give P values as exact values whenever suitable.*
☒	☐	For Bayesian analysis, information on the choice of priors and Markov chain Monte Carlo settings
☒	☐	For hierarchical and complex designs, identification of the appropriate level for tests and full reporting of outcomes
☐	☒	Estimates of effect sizes (e.g. Cohen's *d*, Pearson's *r*), indicating how they were calculated

Our web collection on statistics for biologists contains articles on many of the points above.

Software and code

Policy information about availability of computer code

Data collection	SQL server querying EHR database (made available on PheKB: https://phekb.org/phenotype/chronic-kidney-disease)
Data analysis	R, PLINK, PheWAS, LDSC, RIFTHER, SOLARStrap (all published and freely available)

For manuscripts utilizing custom algorithms or software that are central to the research but not yet described in published literature, software must be made available to editors and reviewers. We strongly encourage code deposition in a community repository (e.g. GitHub). See the Nature Research guidelines for submitting code & software for further information.

Data

Policy information about availability of data

All manuscripts must include a data availability statement. This statement should provide the following information, where applicable:

- Accession codes, unique identifiers, or web links for publicly available datasets
- A list of figures that have associated raw data
- A description of any restrictions on data availability

CKD algorithm and SQL implementation are available from https://phekb.org/phenotype/chronic-kidney-disease; all figures and tables are available from the main and supplement manuscript. The eMERGE-III genetic datasets with linked phenotypes are accessible through dbGAP (accession number: phs001584.v1.p1). The raw clinical data used for developing the algorithms cannot be shared with the public.

Field-specific reporting

Please select the one below that is the best fit for your research. If you are not sure, read the appropriate sections before making your selection.

☒ Life sciences ☐ Behavioural & social sciences ☐ Ecological, evolutionary & environmental sciences

For a reference copy of the document with all sections, see nature.com/documents/nr-reporting-summary-flat.pdf

Life sciences study design

All studies must disclose on these points even when the disclosure is negative.

Sample size	This study used EHR-based clinical data for electronic phenotyping of CKD. All the patients with at least one Cr value in our Clinical Data Warehouse were used for the reported CKD analyses (comorbidity, heritablity). The sample selected for manual review of CKD phenotype was representative of all NKF stages, but selected at random from the output of the CKD algorithm. This study also utilized all available genetic data for GWAS-PheWAS analysis across the eMERGE network.
Data exclusions	We excluded individuals that could not be staged by the algorithm from the analysis of comorbidities and heritability.
Replication	The performance of the CKD algorithm was validated using multiple methods and datasets as described in the manuscript. We additionally evaluated A-stage classifiers by comparing their performance to the recently published methods by Sumida et al. using an independent testing dataset and excluding any data that were used for the development of the classifiers.
Randomization	NA
Blinding	NA

Reporting for specific materials, systems and methods

We require information from authors about some types of materials, experimental systems and methods used in many studies. Here, indicate whether each material, system or method listed is relevant to your study. If you are not sure if a list item applies to your research, read the appropriate section before selecting a response.

Materials & experimental systems

n/a	Involved in the study	
☒	☐	Antibodies
☒	☐	Eukaryotic cell lines
☒	☐	Palaeontology and archaeology
☒	☐	Animals and other organisms
☒	☐	Human research participants
☐	☒	Clinical data
☒	☐	Dual use research of concern

Methods

n/a	Involved in the study	
☒	☐	ChIP-seq
☒	☐	Flow cytometry
☒	☐	MRI-based neuroimaging

Clinical data

Policy information about clinical studies

All manuscripts should comply with the ICMJE guidelines for publication of clinical research and a completed CONSORT checklist must be included with all submissions.

Clinical trial registration	NA
Study protocol	The CKD algorithm development (including A-classifiers) is described in the PheKB documents and in the manuscript methods. The studies of comorbidity, GWAS-PheWAS and heritability are described in the main manuscript methods and the supplement. We use only existing observational EHR data and we do not recruit new patients or collect new variables from participants as part of this study.
Data collection	The data used for CKD patients analysis (CKD phenotyping, comorbidity, heritability) consists of all CUIMC EHR-based clincial data collected before 2016. The validation studies utilize CUIMC, UMN and VU clinical data before 2016. The comparison of A-classifier with the newly published Sumida et al. model utilized more recent non-overlapping CUIMC data (2016-2020). The genetic data for GWAS-PheWAS analysis consists of eMERGE-III genetic datasets with linked phenotypes accessible through dbGAP (accession number: phs001584.v1.p1). No additional data collection was performed as part of this study.
Outcomes	NA
