# Supplementary material for: Medical records-based chronic kidney disease phenotype for clinical care and “big data” observational and genetic studies
Source: NPJ Digit Med. 2021 Apr 13;4:70. doi: 10.1038/s41746-021-00428-1 (PMC8044136; doi:10.1038/s41746-021-00428-1)
Supplement: Supplementary file 2 — Supplementary Information [file 41746_2021_428_MOESM2_ESM.pdf]

Supplemental Data

Medical Records-based Chronic Kidney Disease Phenotype for Clinical Care and "Big Data" Observational and Genetic Studies

Ning Shang, Atlas Khan, Fernanda Polubriaginof, Francesca Zanoni, Karla Mehl, David Fasel, Paul E. Drawz, Robert J. Carrol, Joshua C. Denny, Matthew A. Hathcock, Adelaide M. Arruda-Olson, Peggy L. Peissig, Richard A. Dart, Murray H Brilliant, Eric B. Larson, David S. Carrell, Sarah Pendergrass, Shefali Setia Verma, Marylyn D. Ritchie, Barbara Benoit, Vivian S. Gainer, Elizabeth W. Karlson, Adam S. Gordon, Gail P. Jarvik, Ian B. Stanaway, David R. Crosslin, Sumit Mohan, Iuliana Ionita-Laza, Nicholas P. Tatonetti, Ali G. Gharavi, George Hripcsak, Chunhua Weng, and Krzysztof Kiryluk

Supplementary Table 1. Performance of UPCR-based A-stage classifiers across all discovery and validation cohorts. The performance was generally similar regardless of the dataset used; 95% confidence intervals were calculated based on 10-fold cross-validation applied to the discovery cohorts; the Columbia University (CU) dataset consisted of 4,641 paired UPCR-UACR measurements; the Vanderbilt University (VU) dataset consisted of 5,770 paired UPCR-UACR measurements; the University of Minnesota (UMN) dataset consisted of 8,688 paired UPCR-UACR measurements.

**Model A**		**Model A: UPCR-based classifier using CU data for discovery**		
		**CU Discovery**	**VU Validation**	**UMN Validation**
Squared Error		0.198 (0.197, 0.199)	0.248	0.213
Accuracy (95%CI)	A1	90.3% (89.4%, 91.1%)	81.1%	87.7%
	A2	81.2% (80.0%, 82.5%)	77.2%	81.1%
	A3	90.5% (89.7%, 91.2%)	95.1%	92.2%
Sensitivity (95%CI)	A1	86.4% (84.0%, 88.8%)	79.3%	75.1%
	A2	68.8% (66.3%, 71.2%)	68.8%	70.8%
	A3	86.9% (85.4%, 88.5%)	79.9%	92.4%
Specificity (95%CI)	A1	92.0% (91.0%, 93.1%)	83.9%	92.7%
	A2	87.1% (86.1%, 88.0%)	80.1%	85.9%
	A3	92.5% (91.8%, 93.2%)	97.4%	92.1%

**Model B**		**Model B: UPCR-based classifier using VU data for discovery**		
		**VU Discovery**	**CU Validation**	**UMN Validation**
Squared Error		0.225 (0.217, 0.234)	0.238	0.235
Accuracy (95%CI)	A1	83.9% (83.2%, 84.6%)	87.4%	85.9%
	A2	79.5% (78.5%, 80.5%)	77.1%	78.7%
	A3	94.6% (94.2%, 95.0%)	89.2%	91.7%
Sensitivity (95%CI)	A1	92.3% (91.6%, 93.1%)	93.7%	86.4%
	A2	49.5% (47.0%, 52.1%)	53.9%	57.8%
	A3	73.5% (69.9%, 77.1%)	82.3%	88.8%
Specificity (95%CI)	A1	70.6% (68.8%, 72.3%)	84.6%	85.7%
	A2	89.7% (88.7%, 90.8%)	87.8%	88.6%
	A3	97.7% (97.4%, 98.0%)	93.3%	93.5%

**Model C**		**Model C: UPCR-based classifier using UMN data for discovery**		
		**UMN Discovery**	**CU Validation**	**VU Validation**
Squared Error		0.216 (0.206, 0.226)	0.213	0.239
Accuracy (95%CI)	A1	87.8% (87.3%, 88.2%)	89.7%	82.2%
	A2	80.7% (80.3%, 81.1%)	79.7%	78.0%
	A3	91.8% (91.3%, 92.3%)	89.5%	94.8%
Sensitivity (95%CI)	A1	78.4% (76.5%, 80.3%)	88.3%	82.7%
	A2	70.3% (69.2%, 71.4%)	66.4%	65.9%
	A3	89.5% (88.7%, 90.2%)	83.1%	75.4%
Specificity (95%CI)	A1	91.5% (90.8%, 92.2%)	90.3%	81.4%
	A2	85.6% (85.0%, 86.2%)	85.8%	82.1%
	A3	93.3% (92.4%, 94.1%)	93.2%	97.7%

Supplementary Table 2. Performance of the final UPCR-based A-stage classifier designed by pooling data across all available cohorts; a total of 19,099 paired measurements were used to derive the final model; 95% confidence intervals were calculated based on 10-fold cross-validation applied to the pooled cohort; the performance of the pooled model was tested based on data from each individual institution.

**Test**		**Performance of the pooled UPCR-based A-classifier**			
		**CU + UMN + VU (Pooled)**	**CU**	**UMN**	**VU**
Squared Error		0.219 (0.213, 0.224)	0.215	0.218	0.222
Accuracy (95%CI)	A1	86.7% (86.4%, 87.0%)	89.1%	87.4%	83.8%
	A2	80.0% (79.7%, 80.3%)	79.5%	80.4%	79.7%
	A3	92.3% (92.0%, 92.6%)	89.9%	91.9%	94.9%
Sensitivity (95%CI)	A1	86.2% (85.2%, 87.1%)	90.6%	81.5%	87.6%
	A2	63.5% (62.4%, 64.5%)	62.0%	65.9%	60.1%
	A3	86.7% (85.9%, 87.5%)	84.4%	90.2%	76.6%
Specificity (95%CI)	A1	87.1% (86.4%, 87.7%)	88.4%	89.7%	77.7%
	A2	87.1% (86.6%, 87.5%)	87.6%	87.3%	86.3%
	A3	94.8% (94.5%, 95.1%)	93.1%	93.1%	97.6%

Supplementary Table 3. Performance of the UA-based A-stage classifiers (DSP Scale 1). These models were designed for the DSP Scale 1 (Negative, Trace, 1+, 2+, 3+, 4+) based on the Columbia University (CU) dataset of 12,185 paired UACR-DSP measurements; the performance was tested and 95% confidence intervals (95%CIs) were estimated using a 10-fold cross-validation procedure. The model incorporating urine specific gravity had lower squared validation error and superior accuracy across all A-stages.

**Test**		**Model 1**<br>**A-stage ~ DSP**	**Model 2**<br>**A-stage ~ DSP + SG**
Squared Error		0.287 (0.278, 0.296)	0.256 (0.251, 0.261)
Accuracy (95%CI)	A1	79.6% (78.9%, 80.2%)	80.9% (80.5%, 81.3%)
	A2	75.2% (74.6%, 75.9%)	76.0% (75.6%, 76.4%)
	A3	93.7% (93.5%, 93.9%)	94.3% (94.1%, 94.4%)
Sensitivity (95%CI)	A1	95.4% (94.8%, 96.0%)	90.9% (89.9%, 91.9%)
	A2	29.7% (28.4%, 31.0%)	41.4% (40.1%, 42.8%)
	A3	80.9% (79.7%, 82.2%)	83.3% (82.1%, 84.4%)
Specificity (95%CI)	A1	61.5% (60.3%, 62.8%)	69.6% (68.6%, 70.5%)
	A2	93.0% (92.4%, 93.6%)	89.5% (88.7%, 90.2%)
	A3	96.6% (96.3%, 97.0%)	96.8% (96.5%, 97.1%)

Supplementary Table 4. The performance of the UA-based A-stage classifier (DSP Scale 2). These models were designed for the DSP Scale 2 (Negative, Trace, 10, 30, 100, 300, or >=300). Two datasets of paired UACR-DSP measurements were analyzed, 35,891 from the University of Minnesota (UMN) and 7,595 from Vanderbilt University (VU). We compared the model based on DSP alone (Model 1, M1) to the model based on DSP and urine specific gravity (Model 2, M2). For the purpose of comprehensive testing, each dataset was used separately for model building (with a 10-fold cross-validation) and the other dataset was used for external validation. The performance of the models was generally comparable between the two institutions; the model incorporating urine specific gravity (SG) had consistently lower squared validation error and highest accuracy across all A-stages.

**Test**		**M1 from UMN data A-stage ~ DSP**		**M1 from VU data A-stage ~ DSP**		**M2 from UMN data A-stage ~ DPS + SG**		**M2 from VU data A-stage ~ DPS + SG**	
		**UMN**	**VU**	**VU**	**UMN**	**UMN**	**VU**	**VU**	**UMN**
Squared Error		0.272 (0.266, 0.278)	0.226	0.227 (0.218, 0.235)	0.281	0.244 (0.24, 0.248)	0.204	0.189 (0.182, 0.196)	0.248
Accuracy (95 CI%)	A1	80.0% (79.6%, 80.4%)	82.0%	81.9% (81.1%, 82.8%)	79.6%	81.7% (81.4%, 82.1%)	83.2%	84.6% (83.9%, 85.3%)	81.6%
	A2	75.8% (75.5%, 76.2%)	79.3%	79.3% (78.5%, 80.1%)	76.0%	77.8% (77.4%, 78.2%)	80.6%	82.0% (81.3%, 82.6%)	77.8%
	A3	94.3% (94.1%, 94.5%)	96.4%	96.4% (95.9%, 96.9%)	94.3%	95.0% (94.7%, 95.3%)	96.9%	97.0% (96.7%, 97.2%)	94.9%
Sensitivity (95 CI%)	A1	91.1% (90.8%, 91.4%)	97.0%	97.1% (96.7%, 97.5%)	95.5%	92.0% (91.6%, 92.3%)	96.5%	95.7% (95.2%, 96.1%)	92.2%
	A2	36.4% (35.6%, 37.2%)	25.6%	25.0% (22.4%, 27.6%)	24.5%	36.9% (35.6%, 38.1%)	30.7%	38.2% (35.3%, 41.1%)	34.7%
	A3	69.2% (68.3%, 70.1%)	61.5%	61.7% (58.2%, 65.3%)	69.2%	81.9% (80.8%, 83.1%)	71.9%	77.0% (74.6%, 79.4%)	83.6%
Specificity (95 CI%)	A1	60.8% (60.1%, 61.5%)	45.4%	45.0% (43.0%, 47.0%)	52.2%	64.0% (63.1%, 64.9%)	50.8%	57.5% (55.3%, 59.7%)	63.2%
	A2	88.6% (88.3%, 88.9%)	94.3%	94.4% (93.9%, 95.0%)	92.6%	91.1% (90.6%, 91.5%)	94.5%	94.2% (93.6%, 94.7%)	91.7%
	A3	97.8% (97.6%, 97.9%)	99.1%	99.1% (98.9%, 99.3%)	97.8%	96.8% (96.5%, 97.1%)	98.8%	98.6% (98.4%, 98.7%)	96.5%

Supplementary Table 5. The performance of the final UA-based A-stage classifier for DSP Scale 2. The data for DSP Scale 2 (Negative, Trace, 10, 30, 100, 300, or >=300) were pooled between the University of Minnesota (UMN) and Vanderbilt University (VU) sites to build the final model. This dataset consisted of 43,486 paired DSP-UACR measurements. The predictive properties of the classifiers were tested by a 10-fold cross-validation providing 95% confidence intervals (95%CI). The final pooled model was also applied to individual datasets from both contributing institutions. Model 1 is based on DSP alone; Model 2 is based on DSP and urine specific gravity; Model 2 had lower squared error and higher accuracy across all stages.

Test		Pooled model based on DSP Scale 2					
		UMN + VU (Pooled)		UMN		VU	
		Model 1 (A~DSP)	Model 2 (A~DSP+SG)	Model 1 (A~DSP)	Model 2 (A~DSP+SG)	Model 1 (A~DSP)	Model 2 (A~DSP+SG)
Squared Error		0.264 (0.259, 0.27)	0.235 (0.23, 0.241)	0.272	0.244	0.226	0.194
Accuracy (95%CI)	A1	80.4% (80.0%, 80.7%)	82.2% (81.8%, 82.5%)	80.0%	81.8%	82.0%	84.2%
	A2	76.4% (76.1%, 76.8%)	78.5% (78.1%, 79.0%)	75.8%	77.9%	79.3%	81.5%
	A3	94.7% (94.4%, 94.9%)	95.3% (95.1%, 95.5%)	94.3%	95.0%	96.4%	96.9%
Sensitivity (95%CI)	A1	92.2% (92.1%, 92.4%)	93.2% (93.0%, 93.4%)	91.1%	92.0%	97.0%	97.3%
	A2	34.7% (33.7%, 35.7%)	35.7% (34.0%, 37.4%)	36.4%	36.9%	25.6%	32.4%
	A3	68.3% (66.7%, 69.9%)	81.0% (80.0%, 82.0%)	69.2%	82.2%	61.5%	71.9%
Specificity (95%CI)	A1	58.6% (57.8%, 59.4%)	62.1% (61.1%, 63.1%)	60.8%	64.2%	45.4%	52.2%
	A2	89.6% (89.4%, 89.8%)	92.0% (91.9%, 92.2%)	88.6%	91.2%	94.3%	95.2%
	A3	98.0% (97.9%, 98.2%)	97.1% (97.0%, 97.3%)	97.8%	96.8%	99.1%	98.8%

Supplementary Table 6. Comparison of the performance of A-stage classifiers derived in this study to the alternative methods used by Sumida et al. *Annals of Internal Medicine,* 2020. The paired measurements used for this validation are independent of the ones used for the development of A-classifiers.

Test		UPCR-based A-classifier N=13,134 paired measurements			UA-based A-classifier (DSP+SG) N=6,695 paired measurements		
		Present Study	Sumida et al. Crude Model	Sumida et al. Adjusted Model*	Present Study	Sumida et al. Crude Model	Sumida et al. Adjusted Model*
Squared Error		0.233	0.174	0.175	0.300	0.364	0.374
Overall Accuracy		77.1%	83.1%	82.9%	71.4%	66.8%	65.3%
Accuracy	A1	87.5%	91.9%	91.8%	78.0%	73.0%	73.0%
	A2	77.2%	83.2%	83.0%	71.8%	67.9%	66.2%
	A3	89.4%	91.0%	90.9%	92.9%	92.7%	91.4%
Sensitivity	A1	90.2%	79.5%	79.1%	83.3%	84.7%	79.3%
	A2	64.7%	76.3%	76.4%	55.2%	42.4%	48.7%
	A3	83.0%	90.1%	89.8%	80.9%	81.3%	72.4%
Specificity	A1	87.0%	94.3%	94.3%	75.1%	66.7%	69.6%
	A2	85.1%	87.5%	87.2%	83.1%	85.1%	78.0%
	A3	94.8%	91.8%	91.8%	96.9%	96.4%	97.6%

* adjusted for sex, diabetes, and hypertension

Supplementary Table 7. Secondary validation of the algorithm's diagnostic properties of the CKD algorithm. The validations were performed using N=1,136 patients with a visit to a CKD clinic at Columbia University, and N=1,214 healthy women with a pre-natal visit during the same time period without a known diagnosis of CKD: **(a)** the algorithm has specificity of 97%, sensitivity of 87%, PPV 97%, NPV 89%, and F1 measure of 92% for detecting patients attending the CKD clinic; **(b)** breakdown of the diagnoses by case/control status and stage for the pre-natal visit patients demonstrates that the algorithm correctly classifies N=1178 (97%) of patients as controls, and the remaining N=36 (3.0%) of patients have CKD stage 1 (normal renal function, classified as stage 1 based on positive DSP) or stage 2 (mildly decreased renal dysfunction); **(c)** breakdown of the diagnoses by case/control status and stage for the CKD clinic patients demonstrates that the algorithm correctly classifies N=993 (87%) of CKD cases across all stages, and the remaining 143 (23%) of patients were indeterminate, because the available data were insufficient to meet the algorithm's stringent diagnostic criteria. Notably, among the patients attending the CKD clinic, there were no individuals classified as "controls".

a

**CKD algorithm**	**"Gold Standard"**		**Total**
	**CKD Clinic Patient (Presumed CKD Case)**	**Pre-natal Visit Patient (Presumed Healthy Control)**	
CKD	993	36	1029
Non-CKD or Indeterminate	143	1178	1321
Total	1136	1214	2350

b

**Pre-natal Visit Patients (Presumed Healthy Control) N=1,214**		
**CKD Algorithm Diagnosis**	**CKD Algorithm Stage**	**Count**
Control	CKD G1A1-control	1085
Control	CKD G1-control (G1 but missing urine test)	93
Case	CKD Stage 1 (normal renal function)	18
Case	CKD Stage 2 (mildly reduced renal function)	18

c

**CKD Clinic Patient (Presumed CKD Case) N=1,136**		
**CKD Algorithm Diagnosis**	**CKD Algorithm Stage**	**Count**
Case	CKD Stage 1 (normal renal function)	27
Case	CKD Stage 2 (mildly reduced renal function)	90
Case	CKD Stage 3a	199
Case	CKD Stage 3b	210
Case	CKD Stage 4	177
Case	CKD Stage 5	50
Case	ESRD after transplant	166
Case	ESRD on dialysis	74
Indeterminate	Unable to stage due to missing laboratory data, co-occurrence of conditions violating steady state (e.g. AKI), or inability to establish disease chronicity.	143

Supplementary Table 8. Application of the CKD algorithm to the Columbia Clinical Data Warehouse (CDW): The summary of case counts by **(a)** the National Kidney Foundation (NKF) CKD stage, and **(b)** the Kidney Disease Improving Global Outcomes (KDIGO) A-by-G grid.

a

CKD Stage Classification	Total (N=1,365,098)
**CKD G1-control**	233,244
**CKD G1A1-control**	200,282
**CKD Stage 1**	13,930
**CKD Stage 2**	132,607
**CKD Stage 3a**	38,835
**CKD Stage 3b**	20,374
**CKD Stage 4**	9,310
**CKD Stage 5**	4,761
**ESRD after transplant**	13,676
**ESRD on dialysis**	5,839
**Indeterminate**	692,240

b

NKF Stage	A1 (N=284,141)	A2 (N=27,503)	A3 (N=14,908)	No urine tests
**CKD G1-control**	--	--	--	233,244
**CKD G1A1-control**	200,282	0	0	0
**CKD Stage 1**	4,388	5,366	2,331	1,845
**CKD Stage 2**	56,224	11,809	4,136	60,438
**CKD Stage 3a**	14,401	4,561	2,210	17,663
**CKD Stage 3b**	6,455	3,358	2,182	8,379
**CKD Stage 4**	2,072	1,865	2,254	3,119
**CKD Stage 5***	319	544	1,795	2,103

* Excluding dialysis and transplant

Supplementary Table 9. Age and sex-adjusted prevalence of EHR-captured comorbidities by CKD stage. The P-values correspond to the tests for a linear trend between a given comorbidity and CKD severity as defined by NKF stages.

Comorbidity	Control	CKD Stage 1	CKD Stage 2	CKD Stage 3a	CKD Stage 3b	CKD Stage 4	CKD Stage 5	ESRD D/Tx	P (trend)
Deficiency anemias	1.95	6.45	4.01	7.45	8.24	9.40	9.46	21.8	<2e-16***
Hypertension, uncomplicated	2.88	12.7	11.8	17.4	21.5	17.0	5.31	17.4	<2e-16***
Liver disease	0.75	2.28	1.76	1.93	2.58	5.74	0.87	13.9	<2e-16***
Congestive heart failure	0.5	2.33	3.52	11.4	13.8	8.67	4.67	11.6	<2e-16***
Solid tumor without metastasis	2.52	5.47	5.06	6.67	3.81	5.46	1.50	10.1	<2e-16***
Diabetes without chronic complications	1.54	7.48	4.62	9.09	11.4	10.8	3.83	9.36	<2e-16***
Chronic pulmonary disease	6.71	11.4	11.0	10.6	9.33	5.85	4.65	8.27	<2e-16***
Weight loss	0.93	4.02	2.16	3.69	3.32	2.57	3.67	7.91	<2e-16***
Coagulation deficiency	0.56	1.84	1.14	2.30	4.49	0.85	2.37	6.84	<2e-16***
Other neurological disorders	4.74	7.59	7.12	6.26	7.26	2.74	5.08	5.45	<2e-16***
Diabetes with chronic complications	0.20	2.09	1.04	3.77	4.93	6.11	2.67	5.02	<2e-16***
Metastatic cancer	0.82	3.10	1.99	4.74	2.03	2.17	0.53	4.81	<2e-16***
Depression	2.20	6.30	6.50	6.69	7.48	3.78	0.75	4.53	<2e-16***
Valvular disease	0.75	2.05	2.10	3.57	3.94	2.82	1.81	3.60	<2e-16***
Obesity	3.78	6.34	7.12	6.42	6.27	3.76	1.31	3.44	<2e-16***
RA/collagen vascular diseases	0.57	2.66	1.93	2.82	4.31	3.19	2.28	3.02	<2e-16***
Pulmonary circulation disorder	0.45	1.78	1.71	2.72	5.46	2.5	0.97	2.94	<2e-16***
Hypothyroidism	0.64	1.61	2.75	4.5	2.85	1.51	3.61	2.84	<2e-16***
Psychoses	2.44	5.04	6.41	5.62	4.13	3.68	0.76	2.20	<2e-16***
Lymphoma	0.34	0.71	1.11	2.12	2.43	0.57	0.33	2.43	<2e-16***
Peripheral vascular disorder	0.18	0.97	0.60	0.93	1.46	2.01	0.88	2.23	<2e-16***
Paralysis	1.03	3.15	1.34	2.72	2.36	0.98	0.28	1.37	<2e-16***
Drug abuse	0.84	2.81	2.83	3.37	3.9	3.16	0.83	1.29	<2e-16***
HIV and AIDS	0.41	1.71	2.18	3.15	1.67	1.53	0.85	0.91	<2e-16***
Alcohol abuse	0.68	2.30	1.65	1.61	1.24	1.49	0.48	0.83	<2e-16***
Hypertensive heart disease without CHF	0.11	0.46	0.3	0.47	0.57	0.83	0.54	0.63	<2e-16***
Hypertensive encephalopathy	0.00	0.02	0.02	0.03	0.23	0.04	0.09	0.26	<2e-16***
Hypertension in pregnancy	0.02	0.16	0.09	0.22	0.24	0.40	0.03	0.03	3.6e-05***

Supplementary Figures

Supplementary Figure 1. Predicted A-stage probabilities for three different methods of proteinuria quantification: (a) log-transformed urine protein-to-creatinine ratio (UPCR), **(b)** Dipstick protein (DPS) quantified using *Scale 1* by urine specific gravity (SG), **(c)** Dipstick protein (DPS) quantified using *Scale 2* by urine SG.

a

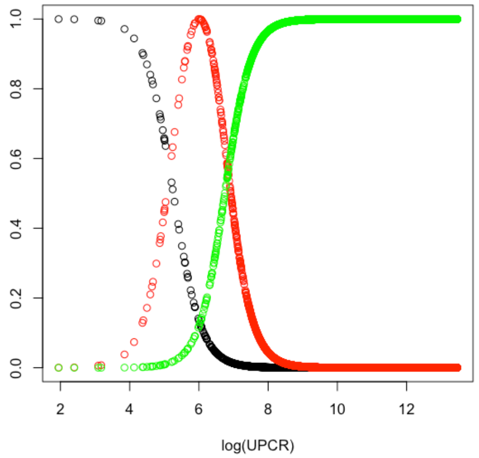

b

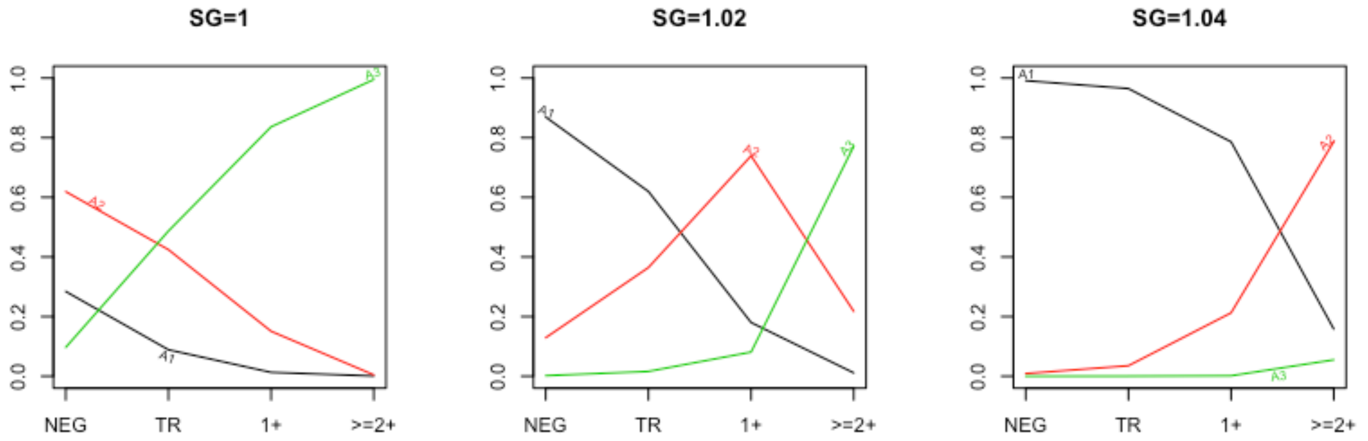

c

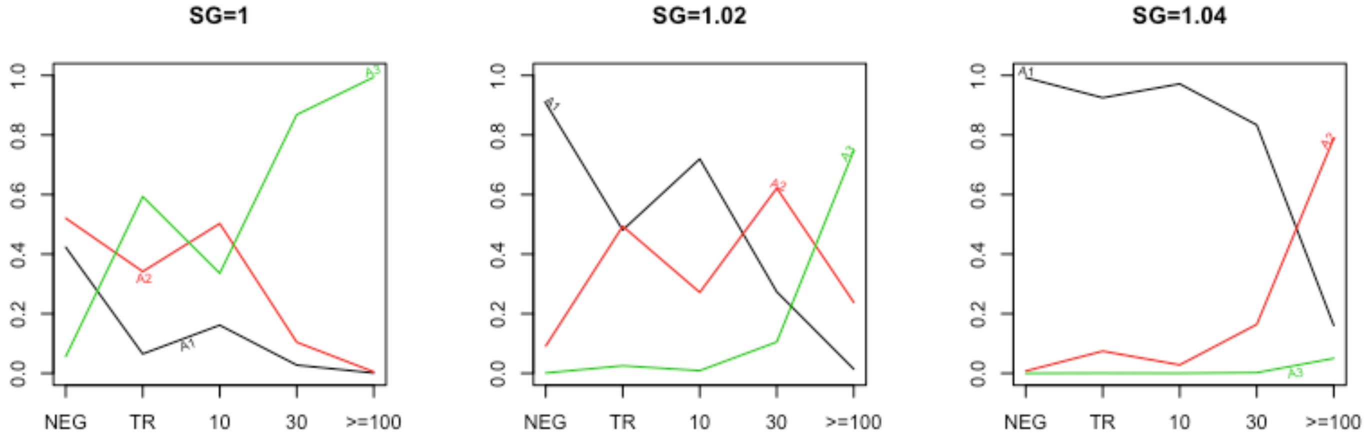

Supplementary Data

Supplementary Data 1. Phenome-wide association study (PheWAS) results for the top SNP at the *UMOD* locus in 78,638 eMERGE participants of genetically defined European-ancestry.

Supplementary Data 2. Phenome-wide association study (PheWAS) results for the top SNP at the *APOL1* locus in 16,976 eMERGE participants of genetically defined African ancestry.

<u>Supplementary Notes</u>

Supplementary Note 1. Final UPCR-based A-stage classifier.

- If UPCR or P24 is 0, then A-staging is classified as A1
- If UPCR or P24 is not 0
  - P(A1)=exp(13.136-2.497*log(UPCR)) / (1+exp(13.136-2.497*log(UPCR)))
  - P(A1,A2)=exp(17.993-2.666*log(UPCR)) / (1+exp(17.993-2.666*log(UPCR)))
  - P(A2) = P(A1,A2) - P(A1)
  - P(A3) = 1- P(A1) - P(A2)
  - A stage = MAX (P(A1), P(A2), P(A3))

Supplementary Note 2. UA-based A-stage classifier (Scale 1 of DPS).

If UA protein data range is (Negative, Trace, 1+, 2+, 3+, 4+)

- If both UA protein and SG are available
    - UA protein is NEGATIVE,
        - P(A1) = exp(-141.736+140.813*SG)/(1+exp(-141.736+140.813*SG))
        - P(A1,A2) = exp(-200.777+203.011*SG)/(1+exp(-200.777+203.011*SG))
    - UA protein is Trace,
        - P(A1) = exp(-143.142+140.813*SG) / (1+exp(-143.142+140.813*SG))
        - P(A1,A2) = exp(-202.959+203.011*SG) / (1+exp(-202.959+203.011*SG))
    - UA protein is 1+
        - P(A1) = exp(-145.145+140.813*SG) / (1+exp(-145.145+140.813*SG))
        - P(A1,A2) = exp(-204.642+203.011*SG) / (1+exp(-204.642+203.011*SG))
    - UA protein is 2+ or more
        - P(A1) = exp(-148.117+140.813*SG) / (1+exp(-148.117+140.813*SG))
        - P(A1,A2) = exp(-208.287+203.011*SG) / (1+exp(-208.287+203.011*SG))
    - P(A2) = P(A1,A2) - P(A1)
    - P(A3) = 1- P(A1) - P(A2)
    - A stage = MAX (P(A1), P(A2), P(A3))
- If only UA protein is available (i.e. the corresponding SG is missing)
    - UA protein of Negative corresponds to A1
    - UA protein of Trace corresponds to A1
    - UA protein of 1+ corresponds to A2
    - UA protein of 2+ or more corresponds to A3

Supplementary Note 3. UA-based A-stage classifier (Scale 2 of DPS).

If UA protein data range is (Negative, Trace, 10, 30, 100, 300, >=300)

- If both UA protein and SG are available
  - UA protein is NEGATIVE
    - P(A1)=exp(-129.764+129.454*SG)/(1+exp(-129.764+129.454*SG))
    - P(A1,A2)=exp(-198.543+201.365*SG)/(1+exp(-198.543+201.365*SG))
  - UA protein is Trace
    - P(A1)= exp(-143.109+140.777*SG)/(1+exp(-143.109+ 140.777*SG))
    - P(A1,A2)= exp(-218.272+231.444*SG)/(1+exp(-218.272+231.444*SG))
  - UA protein is 10
    - P(A1)=exp(-131.101+129.454*SG)/(1+exp(-131.101+129.454*SG))
    - P(A1,A2)=exp(-200.683+201.365*SG)/(1+exp(-200.683+201.365*SG))
  - UA protein is 30
    - P(A1)=exp(-133.02+129.454*SG)/(1+exp(-133.02+129.454*SG))
    - P(A1,A2)=exp(-203.25+201.365*SG)/(1+exp(-203.25+201.365*SG))
  - UA protein is 100 or more
    - P(A1)=exp(-136.286+129.454*SG)/(1+exp(-136.286+129.454*SG))
    - P(A1,A2)=exp(-206.478+201.365*SG)/(1+exp(-206.478+201.365*SG))
  - P(A2) = P(A1,A2) - P(A1)
  - P(A3) = 1- P(A1) - P(A2)
  - A stage = MAX (P(A1), P(A2), P(A3))
- If only UA protein is available (i.e. the corresponding SG is missing)
  - UA protein of Negative corresponds to A1
  - UA protein of Trace corresponds to A1
  - UA protein of 10 corresponds to A1
  - UA protein of 30 corresponds to A2
  - UA protein of 100 or more corresponds to A3
